# *Malat1* regulates myogenic differentiation and muscle regeneration through modulating MyoD transcriptional activity

**DOI:** 10.1038/celldisc.2017.2

**Published:** 2017-03-14

**Authors:** Xiaona Chen, Liangqiang He, Yu Zhao, Yuying Li, Suyang Zhang, Kun Sun, Karl So, Fengyuan Chen, Liang Zhou, Leina Lu, Lijun Wang, Xihua Zhu, Xichen Bao, Miguel A Esteban, Shinichi Nakagawa, Kannanganattu V Prasanth, Zhenguo Wu, Hao Sun, Huating Wang

**Affiliations:** 1 Department of Orthopaedics and Traumatology, Li Ka Shing Institute of Health Sciences, Chinese University of Hong Kong, Hong Kong, China; 2 Department of Chemical Pathology, Li Ka Shing Institute of Health Sciences, Chinese University of Hong Kong, Hong Kong, China; 3 Genome Regulation Laboratory, Guangzhou Institutes of Biomedicine and Health, Chinese Academy of Sciences, Guangzhou, China; 4 RNA Biology Laboratory, RIKEN Advanced Research Institute, Wako, Japan; 5 Department of Cell and Developmental Biology, Chemical and Life Sciences Laboratory, University of Illinois at Urbana-Champaign, Urbana, IL, USA; 6 Division of Life Sciences, The Hong Kong University of Science and Technology, Hong Kong, China

**Keywords:** *Malat1*, miR-181, MyoD, myogenesis, Suv39h1

## Abstract

*Malat1* is one of the most abundant long non-coding RNAs in various cell types; its exact cellular function is still a matter of intense investigation. In this study we characterized the function of *Malat1* in skeletal muscle cells and muscle regeneration. Utilizing both *in vitro* and *in vivo* assays, we demonstrate that *Malat1* has a role in regulating gene expression during myogenic differentiation of myoblast cells. Specifically, we found that knockdown of *Malat1* accelerates the myogenic differentiation in cultured cells. Consistently, *Malat1* knockout mice display enhanced muscle regeneration after injury and deletion of *Malat1* in dystrophic mdx mice also improves the muscle regeneration. Mechanistically, in the proliferating myoblasts, *Malat1* recruits Suv39h1 to MyoD-binding loci, causing trimethylation of histone 3 lysine 9 (H3K9me3), which suppresses the target gene expression. Upon differentiation, the pro-myogenic miR-181a is increased and targets the nuclear *Malat1* transcripts for degradation through Ago2-dependent nuclear RNA-induced silencing complex machinery; the *Malat1* decrease subsequently leads to the destabilization of Suv39h1/HP1β/HDAC1-repressive complex and displacement by a Set7-containing activating complex, which allows MyoD trans-activation to occur. Together, our findings identify a regulatory axis of miR-181a-*Malat1*-MyoD/Suv39h1 in myogenesis and uncover a previously unknown molecular mechanism of *Malat1* action in gene regulation.

## Introduction

Long non-coding RNAs (lncRNAs) are emerging as important regulators of gene expression in major biological processes in the nucleus or cytoplasm, having an impact on cell differentiation, stem cell function and tissue development [1]. As more and more lncRNAs are being functionally characterized [2], a common theme is emerging for nuclear lncRNAs: they drive the formation of ribonucleic–protein complexes with numerous chromatin regulators and then target these enzymatic activities to appropriate locations in the genome, which in turn influence the gene regulation [3–6]. *Malat1* (metastasis-associated lung adenocarcinoma transcript 1) is a lncRNA highly expressed in many tissues and regulated during tissue differentiation. Originally identified as a gene upregulated in metastatic non-small-cell lung cancer cells [7], human *MALAT1* has recently been found overexpressed in many cancers [8, 9]. It localizes to specific nuclear bodies called nuclear speckles [10] where it interacts with several serine- and arginine-rich (SR) family of splicing factors to regulate alternative splicing [11]. In cultured mouse hippocampal neurons, *Malat1* modulates synapse formation by regulating the expression of genes involved in synaptogenesis [12]. Although emerging reports suggest *Malat1* might influence gene expression at transcription level [9], the molecular interactions which allow for such function remain underexplored. RNA pull-down assay followed by mass spectrometry analysis conducted in HeLa cells identified that *MALAT1* could bind to distinctive chromatin modifiers [13]. However, functional characterization of these interactions is still lacking.

In addition to interacting with proteins, emerging evidence indicates frequent association of *MALAT1* with microRNAs. It has become an accepted concept that miRNA–lncRNA interaction is an existing phenomenon in cells [14]. The most well-known mode of the interaction is the competing endogenous model, that is, lncRNAs acting as sponges for miRNAs and competing for miRNAs’ binding to bona fide mRNA targets [15]. However, recent reports support that interaction between *MALAT1* and miRNA often leads to downregulation of *MALAT1* abundancy [16–18]. Nevertheless, the underlying molecular mechanism remains unexplored, especially whether the canonical RISC machinery used for miRNA targeting in the cytoplasm is also utilized for the degradation of nuclear-residing *MALAT1*.

Myogenesis, which occurs in both postnatal growth and the regeneration of skeletal muscle after an injury, is a highly ordered process that can be subdivided into multiple steps. These steps include the activation of satellite cells (SCs or adult muscle stem cells) into committed myoblasts, followed by proliferation and differentiation of myoblasts, resulting in cell fusion to form myotubes [19, 20]. Sequential activation of the muscle-specific transcriptional factors has a crucial role in regulating myogenesis [21]. Owing to the early discovery of the myogenic factors that act downstream, the myoblast differentiation has been extensively studied. It is a powerful system for investigating the biological functions of lncRNAs because it can be partially recapitulated *in vitro* using a C2C12 murine myoblast cell line and because the transcriptional networks coordinating gene expressions are well investigated. Specification and differentiation of myoblasts into myotubes are driven by MyoD, Myf5, Myogenin and MRF4; upon induction by MyoD, Myogenin together with MyoD activates the transcription of muscle-specific genes such as myosin heavy chain (MyHC), alpha actin (α-actin) and troponin isoforms. Recent lines of work from our group [22–26] and others demonstrate that complex networks of transcription factors, chromatin regulators and microRNAs orchestrate tight control of myogenic differentiation through integrating intrinsic and extrinsic input into the gene expression program [21, 27, 28]. Emerging evidence from our group and others also demonstrates that lncRNAs are novel components of these regulatory networks [15, 29, 30]. Of particular interest, two recent studies [31, 32] investigated the function of *Malat1* during myogenesis in C2C12 showing promoting effect of *Malat1* knockdown on C2C12 differentiation. However, the underlying molecular mechanism needs to be further explored and the function of *Malat1* in myogenesis also needs further clarification in a regeneration setting *in vivo*.

When associated to the consensus E-box sequence within muscle-specific gene promoters, MyoD, as a master regulator, can recruit multiple transcriptional regulators and interact with the basal transcriptional machinery to activate gene expression [33]. Chromatin immunoprecipitation-sequencing (ChIP-Seq) analysis has revealed that MyoD binds to a large number of genomic loci in proliferating myoblasts but is unable to activate their transcription [34], reinforcing the notion that MyoD activity must be precisely regulated to orchestrate the transcriptional program. One mechanism is through the association with Suv39h1, a histone H3 lysine 9 (H3-K9) methyltransferase. In the proliferating myoblasts, Suv39h1 is recruited to the MyoD-bound muscle gene loci to suppress their expression through its histone methyltransferase activity [35]; upon differentiation, Suv39h1 is replaced by Set7, which results in activation of the gene expression [36]. In addition to Suv39h1, MyoD also recruits HDACs and HP1β to target promoters, together forming a silencing complex to suppress gene expression by inducing a locally repressive chromatin structure [37]. Still, it is yet to answer the questions: how is Suv39h1 recruited to MyoD-bound loci? How is the complex stabilized in proliferating myoblasts and displaced upon differentiation?

To address the physiological function of *Malat1* in a living organism, three groups have independently generated *Malat1* knockout (KO) mice using homologous recombination [38–40]. Unexpectedly, the *Malat1*-KO mice display no apparent abnormalities, suggesting that *Malat1* is not essential in living mice maintained under normal conditions; its function probably becomes apparent only in specific cell types or under certain stress or pathological conditions. This also demonstrates that our understanding of the *Malat1* function in both physiological and pathological conditions is far from completion. In this project, we thus investigate the function of *Malat1* in skeletal muscle cells and muscle regeneration. Our findings reveal that *Malat1* indeed has a critical role during myoblast differentiation. Knockdown of *Malat1* accelerates the myogenic process. Functional elucidation uncovers a novel molecular mechanism of *Malat1* regulating MyoD trans-activation through recruiting Suv39h1 to the MyoD-binding loci. *In vivo*, the injury-induced muscle regeneration process is accelerated with genetic deletion of *Malat1* owing to the enhanced myogenic capacity of SCs. Lastly, we also demonstrate that *Malat1* transcript is directly targeted by miR-181a and degraded through Ago2-dependent nuclear RNA-induced silencing complex (nRISC) machinery occurring in the myoblast nucleus. Altogether, our results have identified a previously unknown molecular mechanism of *Malat1* action.

## Results

### *Malat1* is dynamically expressed during skeletal muscle differentiation

**Figure 1:**
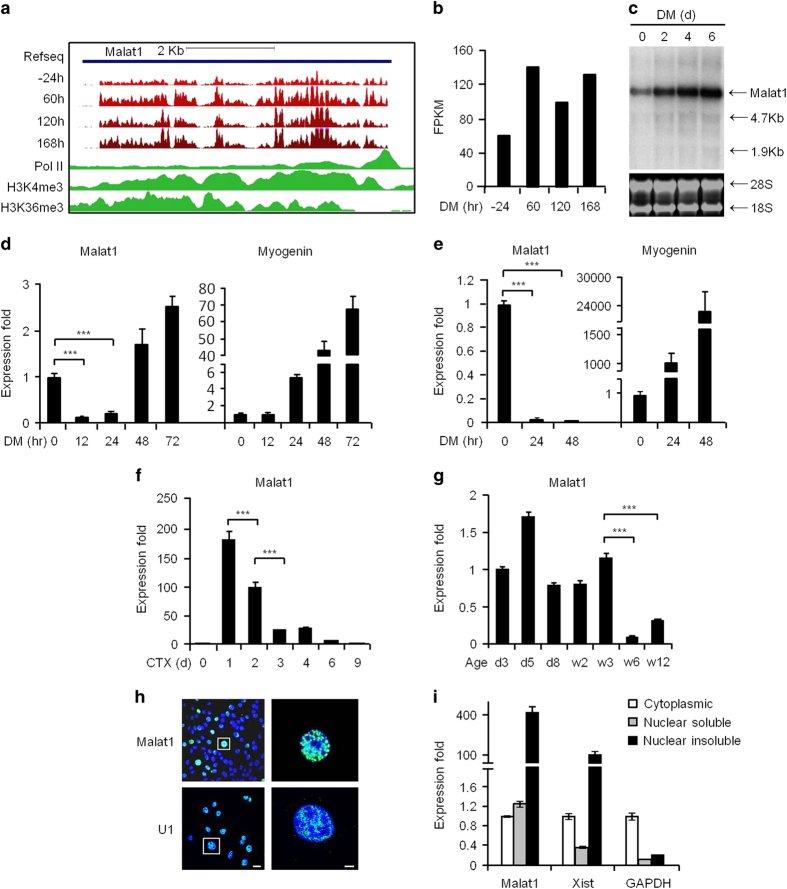
*Malat1* is associated with skeletal myogenesis. (**a**) Genomic snapshot of mouse *Malat1* generated in Refseq (blue), RNA-seq (red, from C2C12 cells in DM for −24, 60, 120 and 168 h), Pol II, H3K4me3 and H3K36me3 ChIP-seq (green) tracks from C2C12 cells in growing medium. (**b**) The Fragments Per Kilobase of transcripts per Million mapped reads (FPKM) values of *Malat1* from the above RNA-seq data. (**c**) The expression of *Malat1* at the indicated time points in DM was detected by northern blotting. 18S and 28S rRNAs are used as loading controls. The size of the ladder was shown on the right. (**d**) The expression of *Malat1* and *Myogenin* during C2C12 differentiation was examined by qRT-PCR. (**e**) The expression of *Malat1* and *Myogenin* in early-differentiating satellite cells freshly isolated from mouse limb muscles. (**f**) The expression of *Malat1* during CTX-induced regeneration at the indicated days post-CTX injection; (**g**) in muscles of postnatal mice at the indicated age (week); (**h**) visualization of *Malat1* or U1 transcripts in C2C12 myoblasts by RNA FISH. The images on the right are the boxed regions in a higher magnification (×1 000). Left panel scale bar=20 μm. Right panel scale bar=5 μm. (**i**) The expression of *Malat1, Xist* and *GAPDH* in three fractions extracted from C2C12 myoblasts: cytoplasmic, nuclear-soluble or nuclear-insoluble fraction. All PCR data were normalized to *GAPDH* mRNA and represent the average of three independent experiments±s.d. ***P<0.001.

To investigate whether *Malat1* is a functional molecule during skeletal muscle differentiation, we first examined its temporal and spatial expression patterns in several myogenesis systems *in vitro* and *in vivo*. When C2C12 myoblasts were induced to differentiate by switching the cells from proliferation to serum-deprived differentiation medium (DM), according to the published high-throughput RNA-sequencing (RNA-seq) data [41], *Malat1* expression was highly expressed in myoblasts and continuously upregulated in a course of 0, 60, 120 and 168 h in DM (Figure 1a and b); this was confirmed by northern blot analysis: a strong signal was detected around 7 kb with the intensity increasing from 0 to 6 days in DM (Figure 1c). However, when taking a closer look at the earlier time points of differentiation (<24 h), *Malat1* was markedly downregulated at DM 12 h (86% decrease) and 24 h (79% decrease), whereas the early-differentiation marker gene, *Myogenin*, was continuously induced (Figure 1d and Supplementary Figure S1a and b), suggesting that *Malat1* may act as an anti-myogenic factor during the early differentiation of C2C12 myoblasts. Consistently, during the differentiation of freshly isolated SCs, *Malat1* was also remarkably decreased (Figure 1e). To further examine its expression dynamics during myogenesis *in vivo*, we employed a widely used muscle regeneration model in which the intramuscular injection of cardiotoxin results in muscle injury and in turn induces muscle regeneration [29, 30]. *Malat1* was found to be highly induced during the early regeneration stage when SCs became activated, proliferated and started to differentiate, but gradually downregulated later on when the newly formed fibers matured and regeneration completed in ~9 days (Figure 1f). Consistently, high level of *Malat1* was observed in limb muscles of newborn mice (aged 3 days to 3 weeks), which displayed active myogenesis, but the level of *Malat1* was decreased as the neonatal myogenesis ceased after ~3 weeks (Figure 1g). Moreover, when compared with normal muscles from wild-type (WT) mice, a higher level of *Malat1* was detected in dystrophic muscles from young mdx mice, a mouse model of Duchenne muscular dystrophy at 3, 5 or 6 weeks of age, which were featured by a pathologically active degeneration–regeneration process (Supplementary Figure S1d). The above results suggested that *Malat1* is associated with active myogenesis processes *in vitro* and *in vivo*.

To gain more functional insights, the subcellular localization of *Malat1* was examined using RNA fluorescence *in situ* hybridization (FISH). In line with the previous reports showing that *Malat1* is located in the nuclear speckles in many cell types [10, 11], *Malat1* was found exclusively in the nucleus of C2C12 myoblast (Figure 1h) and myotube (Supplementary Figure S1b); as a positive control, the nuclear transcript snRNA *U1* was also found restrictively in the nucleus (Figure 1h). A high magnification of the FISH image revealed its focal distribution pattern (Supplementary Figure S1c), resembling the staining pattern of Srsf2, a marker for nuclear speckle, suggesting that a large portion of *Malat1* may localize in the nuclear speckles in C2C12 cells as in many other cell types [42]. By cellular fractionation assay, *Malat1* transcripts were highly enriched in the insoluble fraction of nuclei where the chromatin-binding lncRNA *Xist* was also detected; *GAPDH* transcripts, on the other hand, were mainly found in the cytoplasmic extracts (Figure 1i).

### *Malat1* represses myoblast differentiation

**Figure 2:**
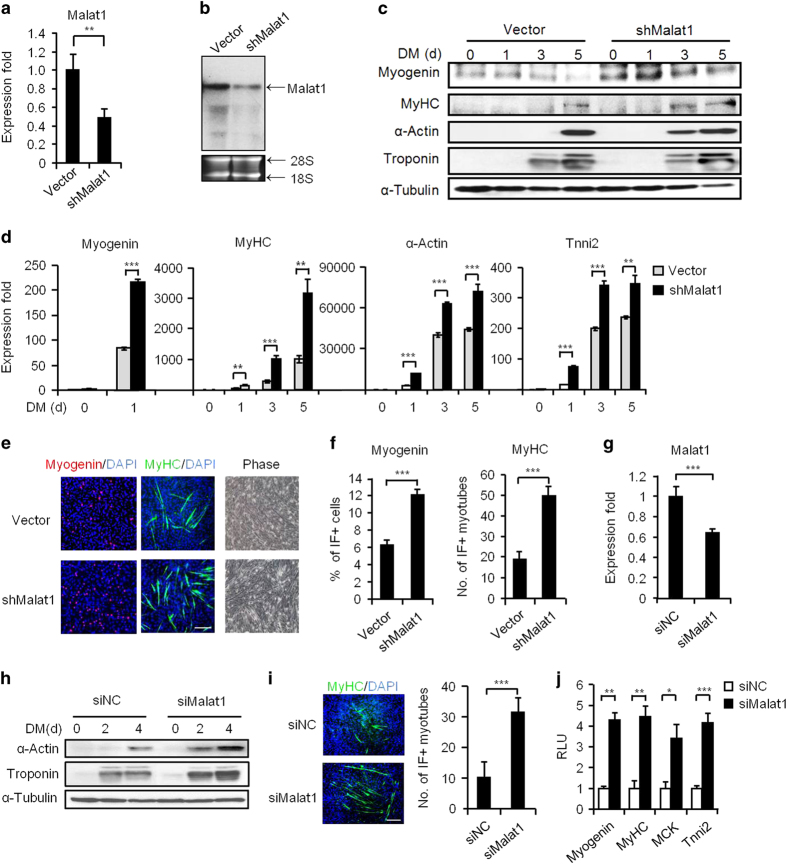
*Malat1* functions to repress myoblast differentiation. (**a**, **b**) The knockdown of *Malat1* in C2C12 cells by a stably expressed shRNA oligo was confirmed by qRT-PCR (**a**) and northern blotting in proliferating myoblasts (**b**). (**c**) The knockdown of *Malat1* increased the levels of the indicated myogenic genes, Myogenin, MyHC, α-Actin and Troponin at protein level during a 5-day differentiation course. (**d**) The knockdown of *Malat1* increased the above myogenic genes at mRNA levels. (**e**) The above cells were visualized on day 2 in DM (phase images). IF staining for Myogenin or MyHC was performed on day 1 and day 3 in DM, respectively. (**f**) The number of positively stained cells was counted from at least 10 fields. (**g**) The knockdown of *Malat1* by a siRNA oligo was confirmed by qRT-PCR on day 2 post transfection. (**h**) The above knockdown increased the levels of the indicated myogenic genes α-Actin and Troponin at the protein level during a 4-day differentiation course. (**i**) The above si*Malat1* transfection also increased the number of MyHC-positive cells by IF staining on day 2 in DM. (**j**) The knockdown of *Malat1* in C2C12 cells increased the luciferase activities of the indicated myogenic reporters on day 2 in DM. RLU, relative luciferase unit. All PCR data were normalized to *GAPDH* mRNA and represent the average of three independent experiments±s.d. All luciferase data were normalized to Renilla luciferase activities and represent the average of three independent experiments±s.d. *P<0.05, **P<0.01, ***P<0.001.

The early downregulation of *Malat1* expression during myoblast differentiation suggested that it might be an anti-myogenic factor in the initial stage of differentiation. To test this notion, we generated a C2C12 cell line stably expressing a short hairpin RNA (shRNA) against *Malat1*. Knockdown of *Malat1* (Figure 2a, 53% knockdown efficiency, and Figure 2b) significantly enhanced C2C12 differentiation as assessed by the examination of several myogenic markers, Myogenin, MyHC, α-Actin and Troponin, at both protein (Figure 2c) and RNA (Figure 2d) levels during a differentiation course of 5 days. The results were also strengthened by immunofluorescence (IF) staining for Myogenin and MyHC proteins in differentiating myotubes on day 1 and 3, respectively (Figure 2e); the number of positively stained myotubes was increased by sh*Malat1* treatment by 1.9- and 2.7-fold, respectively (Figure 2f). To confirm the above results, transient transfection of a short interfering RNA (siRNA) oligo against *Malat1* to C2C12 cells was performed (Figure 2g, 36% knockdown efficiency), and the same accelerating effect was observed by assaying for expression of the myogenic markers (Figure 2h and i). Furthermore, transfection of a myogenic luciferase reporter together with si*Malat1* oligos significantly increased the reporter activities as compared with negative control oligos (Figure 2j, Myog-Luc, 4.3-fold; Tnni2-Luc, 4.2-fold; Muscle creatine kinase, MCK-Luc, 3.4-fold; MyHC-Luc, 4.5-fold). Owing to its large size and high abundance, *Malat1* is notorious for the difficulty in knocking down; we managed to find a second siRNA oligo targeting a different region of *Malat1*, which also led to a similar pro-myogenic phenotype (Supplementary Figure S2h–j). Conversely, increase in the *Malat1* level by directly transfecting *in vitro*-transcribed *Malat1* transcripts suppressed the C2C12 differentiation (Supplementary Figure S2a–c). In addition, *Malat1* appeared to promote C2C12 myoblast proliferation as its knockdown inhibited cell proliferation as assessed by viable cell counting (Supplementary Figure S2d), MTT assay (Supplementary Figure S2e) and 5-bromo-2'-deoxyuridine (BrdU) staining (Supplementary Figure S2f, 10% decrease by si*Malat1*), whereas overexpression of *Malat1* accelerated myoblast proliferation (Supplementary Figure S2g).

### *Malat1* represses the transcriptional activity of MyoD

**Figure 3:**
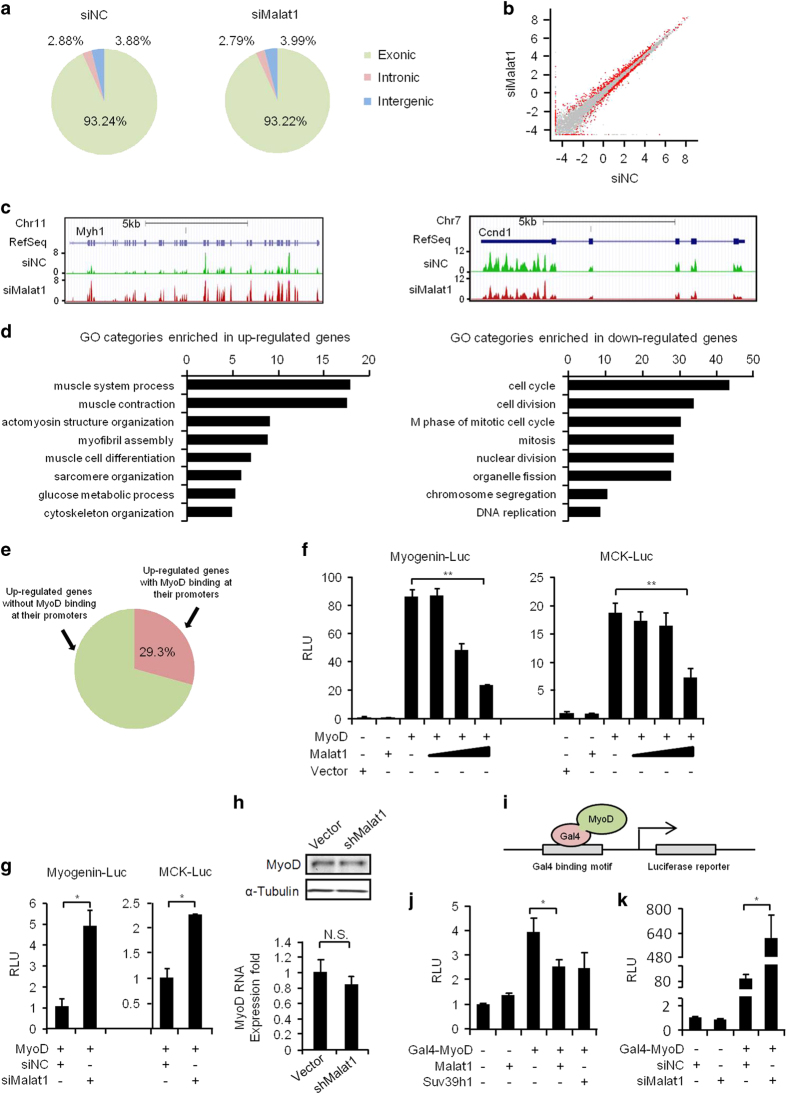
*Malat1* modulates the transcriptional activity of MyoD. (**a**) RNAs with PolyA tails (PolyA^+^ RNAs) were isolated from C2C12 cells transfected with siNC or si*Malat1* oligos and differentiated for 2 days and subjected to RNA-seq for transcriptomic analysis. The normalized fragment density was calculated by counting the fragments per kilobase of genomic regions of interests (exonic, intronic and intergenic) per million mapped reads (FPKM). (**b**) Differentially expressed genes between siNC (*x* axis) and si*Malat1* (*y* axis) cells were determined from the above RNA-seq data and shown as red dots in the scatter plot. Gray dots represent genes unaffected by si*Malat1* treatment. (**c**) Genomic snapshots showing an example of significantly up- (*Myh1*, left) or down- (*Ccnd1*, right) regulated genes by si*Malat1* treatment. The* y* axis is the normalized signal density. (**d**) GO analysis of genes that were up- or downregulated in si*Malat1* cells. The *y *axis shows GO terms and the* x *axis shows statistical significance (that is, −log (p), multicorrected *P*-values) for the top eight enriched terms. (**e**) A high percentage (29.3%; in red color) of the above upregulated genes contain a MyoD-binding site at their promoter regions based on the analysis of MyoD ChIP-seq data from C2C12 cells. (**f**) 10T1/2 cells were co-transfected with a Myogenin or MCK luciferase reporter, a MyoD expression plasmid (0.5 μg) and a *Malat1* expression plasmid (0, 0.125, 0.25 or 0.5 μg). Luciferase activities were measured after differentiating the cells for 48 h. (**g**) 10T1/2 cells were transfected with the indicated reporter, the MyoD plasmid and the siRNAs against *Malat1* or negative control (siNC) oligos. Luciferase activities were measured after differentiating the cells for 48 h. (**h**) Knockdown of *Malat1* did not affect the expression of MyoD at protein (upper, western blotting) or RNA (bottom, qRT-PCR) level. (**i**) Schematic illustration of the Gal4-MyoD reporter system. A Gal4-binding motif is fused to the luciferase gene; the expression of a fusion protein with Gal4-binding domain fused with MyoD (Gal4-MyoD) will lead to its binding to the Gal4 motif and activate the luciferase reporter. (**j**) 10T1/2 cells were co-transfected with the above Gal4 luciferase reporter, the *Malat1* expression vector (0.5 μg) or a Suv39h1 expression plasmid in the presence of the vector expressing Gal4-MyoD fusion protein. Luciferase activities were measured after differentiating the cells for 48 h. (**k**) 10T1/2 cells were co-transfected with the Gal4 luciferase reporter, si*Malat1* or siNC oligos and the Gal4-MyoD vector. Luciferase activities were measured after differentiating the cells for 48 h. All PCR data were normalized to *GAPDH* mRNA and represent the average of three independent experiments±s.d. All luciferase data were normalized to Renilla luciferase activities and represent the average of three independent experiments±s.d. *P<0.05, **P<0.01.

To gain insights into molecular mechanisms of how *Malat1* regulates the myogenic differentiation, we conducted an RNA-seq analysis to globally characterize the transcriptomic changes affected by *Malat1*. Polyadenylated RNAs were extracted from C2C12 cells transfected with si*Malat1* or siNC oligos and subjected to RNA-seq analysis (Figure 3a and Supplementary Figure S3a and b). As a result, a total of 427 genes were upregulated, whereas 403 were downregulated (Figure 3b and c and Supplementary Table S1), indicating that *Malat1* knockdown induced a global transcriptomic change in the muscle cell. Subsequent GO analysis revealed that the upregulated genes were enriched for muscle-related terms (Figure 3d, left). The downregulated genes, on the other hand, were enriched for cell cycle-related terms (Figure 3d, right). This was consistent with the enhanced differentiation and inhibited proliferation phenotype that we observed in Figure 2. Many of the upregulated genes were muscle genes known to be regulated by the muscle master transcription factor, MyoD, leading us to suspect that *Malat1*’s function may be associated with MyoD regulation. Indeed, by exploring MyoD ChIP-Seq data from C2C12 cells [34], we found that almost 30% of above upregulated genes had MyoD binding at their promoters (Figure 3e).

To further test *Malat1* regulation of MyoD activity, C3H/10T1/2 (10T1/2) fibroblasts were co-transfected with a MyoD expression plasmid and a MyoD-responsive luciferase reporter (Myogenin-Luc or MCK-Luc) in the presence of an increasing amount (0, 0.125, 0.25 or 0.5 μg) of *Malat1*-encoding plasmid. The results from luciferase assays demonstrated that *Malat1* inhibited the MyoD trans-activation of these reporters in a dose-dependent manner (Figure 3f; Supplementary Figure S3c and e). On the contrary, transfection of si*Malat1* oligos led to enhanced activation of the myogenic reporters by MyoD (Figure 3g; Supplementary Figure S3d and f). Altogether, the above data suggested that *Malat1* regulates myogenic differentiation through modulating MyoD activity. Interestingly, sh*Malat1* knockdown did not affect the expression of MyoD itself (Figure 3h). To further examine whether *Malat1* regulates the trans-activation function of MyoD, we employed a Gal4 luciferase reporter system. As illustrated in Figure 3i, a Gal4-binding motif is fused to the luciferase gene; the expression of a fusion protein with Gal4 DNA-binding domain fused with MyoD (Gal4-MyoD) will lead to its binding to the Gal4 motif and activate the luciferase reporter. 10T1/2 cells were co-transfected with the above Gal4 luciferase reporter and the Gal4-MyoD fusion plasmid in the presence or absence of the *Malat1*-expressing plasmid. Trans-activation of the reporter was found to be inhibited by *Malat1* expression (Figure 3j and Supplementary Figure S3g). Ectopic expression of Suv39h1, as previously shown [35], also inhibited the MyoD trans-activation (Figure 3j). Conversely, co-transfection of si*Malat1* oligos enhanced the MyoD trans-activation in the assay (Figure 3k and Supplementary Figure S3h–j). These results suggested that *Malat1* could regulate the trans-activating ability of MyoD.

### *Malat1* recruits Suv39h1 to the MyoD-binding loci

**Figure 4:**
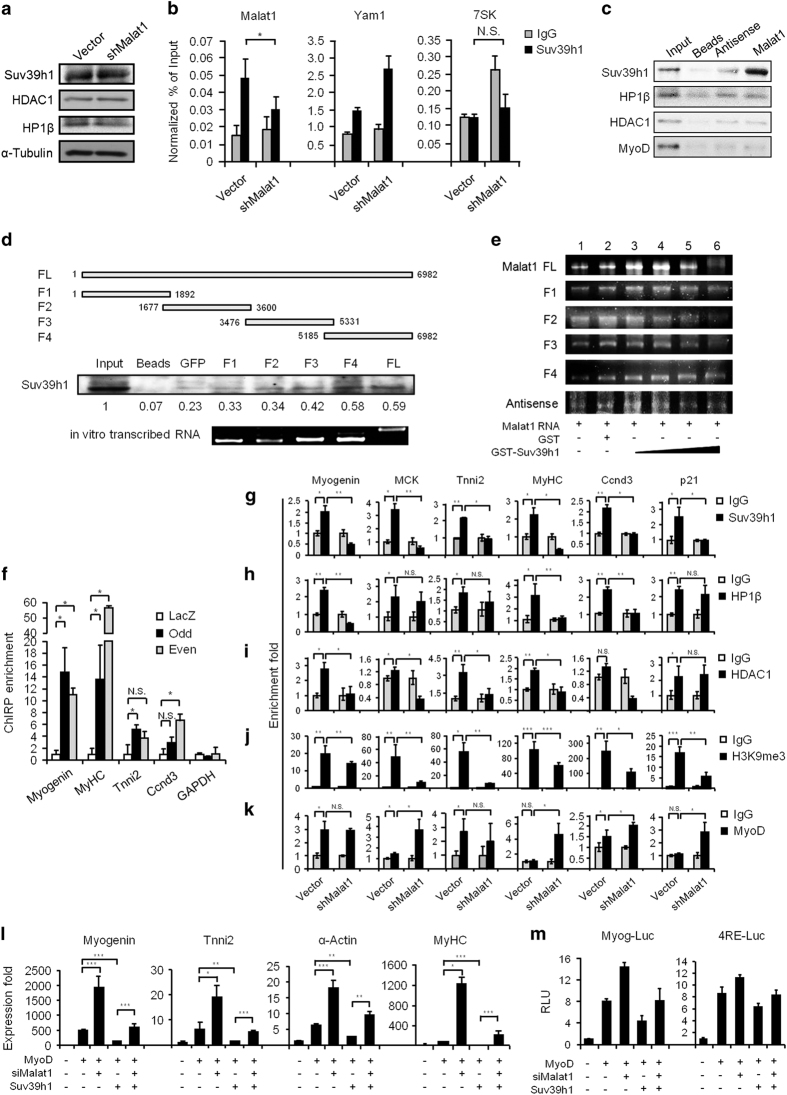
*Malat1* interacts and recruits Suv39h1 to MyoD-binding loci. (**a**) The expression levels of Suv39h1, HDAC1 and Hp1β proteins in Vector or sh*Malat1* C2C12 cells were determined by western blotting in proliferating myoblasts. α-Tubulin was used as a normalization control. (**b**) Nuclear extracts from the above cells were immunoprecipitated by IgG or an antibody against Suv39h1. The retrieved RNAs were detected by qRT-PCR. (**c**) *In* *vitro*-transcribed biotinylated *Malat1* FL transcripts were used to pull down its binding proteins. The indicated proteins were detected by western blotting. Beads only or antisense *Malat1* transcripts were used as negative controls. (**d**) Upper: schematic illustration of the *Malat1* FL or fragments (F1, F2, F3 or F4) that were used in the RNA pull-down assays for mapping the functional domain. Beads only or GFP transcripts were used as negative controls. Middle: the retrieved Suv39h1 proteins were detected by western blotting. The numbers below indicate the quantification of the intensity of each band. Lower: the *in vitro* generated transcripts were visualized on an agarose gel. (**e**) EMSA assays were performed to detect the association between *Malat1* FL or various fragments with purified GST-Suv39h1 protein (for 200 fmol *Malat1* FL RNA, 100, 400, 800 or 1 600 nM GST-Suv39h1 were used. For 400  fmol F1–F4 fragments, 800, 1 600, 2 400 or 3 200 nM GST-Suv39h1 protein were used). Antisense *Malat1* transcript was used as an unrelated negative control for RNA. GST only protein (3 200 nM) was used as a negative control for GST-Suv39h1 protein. A shifted band (lanes 5 and 6) ran higher than the control bands (lanes 1 and 2) and also displayed lower intensity. (**f**) *Malat1* ChIRP with both even and odd antisense oligos retrieved a significant amount of genomic DNAs corresponding to the promoter regions of *Myogenin*, *MyHC*, *Tnni2* and *Ccnd3* genes but not *GAPDH* locus. LacZ ChIRP retrieved no signal. (**g**–**k**) ChIP-PCR analysis of Suv39h1, HP1β, HDAC1, H3K9me3 and MyoD enrichment on the promoter or enhancer of *Myogenin*, *MCK*, *MyHC*, *Troponin*, *Ccnd3* and *p21* loci in Vector or sh*Malat1* expressing C2C12 cells. The enrichment fold was calculated as a fraction of DNA present in the input samples. (**l**) 10T1/2 cells were transfected with the indicated combination of plasmids and si*Malat1* oligos. The expression of the myogenic marker genes was analyzed by qRT-PCR. (**m**) 10T1/2 cells were co-transfected with a Myogenin or 4RE luciferase reporter, the indicated expression plasmids and si*Malat1* oligos. Luciferase activities were measured after differentiating the cells for 48 h. All PCR data were normalized to *GAPDH* mRNA and represent the average of three independent experiments±s.d. All luciferase data were normalized to Renilla luciferase activities and represent the average of three independent experiments±s.d. *P<0.05, **P<0.01, ***P<0.001.

To further investigate how *Malat1* inhibits the trans-activating ability of MyoD, we thought of the possibility that *Malat1* could recruit a repressive cofactor to the MyoD-binding loci. As *Malat1* had been shown to interact with Set domain-containing proteins [13], we hypothesized that *Malat1* might interact with Suv39h1 and recruit it to MyoD. To test this notion, we need to first exclude the possibility that *Malat1* modulates Suv39h1 activity through regulating its expression. Indeed, the levels of Suv39h1 and its partners in the repressive complex, HP1β, and HDAC1, were not affected by sh*Malat1* treatment in C2C12 myoblasts (Figure 4a). Next, using crosslinked RNA immunoprecipitation (RIP) assay, antibodies against Suv39h1 protein retrieved a significant amount of endogenous *Malat1* transcripts from C2C12 myoblasts (3.2-fold enrichment compared with IgG pull-down; Figure 4b), whereas *Yam1* [29] and *7SK* [43] were not retrieved, indicating a putative interaction between Suv39h1 protein and *Malat1* transcripts. To strengthen this finding, we then further performed RNA pull-down assay from native non-crosslinked cell lysates using biotinylated *in vitro*-transcribed full-length (FL) *Malat1* transcripts. Consistently, *Malat1* transcripts pulled down a substantial amount of endogenous Suv39h1 protein; however, no HP1β, HDAC1 or MyoD protein was retrieved (Figure 4c), suggesting a direct physical interaction between Suv39h1 and *Malat1*. As negative controls, beads alone, green fluorescent protein (GFP) transcripts or antisense *Malat1* transcripts did not retrieve Suv39h1 protein either (Figure 4c). To further map the binding domain, a series of deletion mutants of *Malat1* was tested for the binding with Suv39h1. Interestingly, the 3′ domain (5 185–6 982  nt, F4) alone retrieved almost the equal amount of Suv39h1 as the FL transcript; fragments F2 and F3 also exhibited some binding activity, whereas fragment F1 had very low binding with Suv39h1 (Figure 4d). Similar results were obtained when a Flag-tagged Suv39h1 was expressed in C2C12 cells and used in the assay (Supplementary Figure S4a). To further confirm the direct binding between *Malat1* and Suv39h1, we performed *in vitro* RNA–protein electrophoretic mobility shift assay (EMSA). Purified GST (glutathione *S*-transferase)-Suv39h1 protein and *in vitro*-transcribed *Malat1* full length or fragments were incubated and analyzed for their binding on a non-denaturing agarose gel. As shown in Figure 4e, a gradual shift of *Malat1* FL RNAs was observed as the concentration of GST-Suv39h1 increases (from lanes 3 to 6); 1 600 nM yielded the most evident shift (lane 6; see also Supplementary Figure S4b); GST protein alone (Figure 4e, lane 2) or a GST-MyoD protein (Supplementary Figure S4c) did not cause the shift. The above results confirmed the physical association between *Malat1* FL and Suv39h1. In addition, consistent with the results from RNA pull-down assay in Figure 4d, GST-Suv39h1 protein was able to cause an evident shift of *Malat1* F4 fragment and a relatively weaker shift of F2 and F3; such a shift appeared non-existent for F1 or negative control antisense *Malat1* (Figure 4e). When further mapping the interacting domain in Suv39h1 protein [44], we found that SET/Post-SET domain that catalyzes histone methyltransferase activity displayed evident binding with *Malat1*; Pre-SET domain or Chromodomain (chromatin-binding domain), whereas displayed no binding with *Malat1* (Supplementary Figure S4d). Next, to answer the question whether *Malat1* is tethered to the MyoD-binding DNA loci, Chromatin Isolation by RNA Purification (ChIRP) assay [45] with both odd and even tiling oligos against *Malat1* was performed. We were able to specifically retrieve substantial amount of endogenous *Malat1* transcripts from the promoter regions of MyoD/Suv39h1 target genes including *Myogenin*, *Tnni2*, *MyHC* and *Ccnd3* (Figure 4f and Supplementary Figure S4e). Next, we performed ChIP-PCR assays to show that knockdown of *Malat1* significantly impaired the binding of endogenous Suv39h1 protein to the MyoD-binding loci, including *Myogenin*, *MCK*, *Tnni2*, *MyHC*, *Ccnd3* and *P21* promoter or enhancer (Figure 4g, Supplementary Figure S4f), confirming that *Malat1* was critical for the recruitment of Suv39h1 to the MyoD target loci. In addition, the occupancy of HP1β and HDAC1 was also decreased on some of the above loci in the sh*Malat1* C2C12 cells (Figure 4h and i), even though *Malat1* did not directly interact with them, suggesting that *Malat1* may also affect the stability of the repressive complex. Suv39h1 is a methyltransferase that induces trimethylation of histone 3 lysine 9 (H3K9me3) at target sites. Expectedly, H3K9me3 levels at all the above target loci were significantly reduced upon *Malat1* knockdown (Figure 4j). Interestingly, occupancy of MyoD on *MCK*, *MyHC* and *Ccnd3* was enhanced after knocking down *Malat1* (Figure 4k), which was probably correlated with the induction of the genes. Although we could only detect increased Set7 occupancy on some sites (Supplementary Figure S4g), there was no significant increase in H3K4me1 despite the role of Set7 as a Histone 3 monomethylase [36], suggesting that the increased transcription following Suv39h1 displacement may be mediated through other mechanisms. Altogether, the above results supported that *Malat1* had a critical role in regulating MyoD trans-activation by recruiting Suv39h1 to the target loci. To further strengthen our findings, we showed that si*Malat1* transfection into 10T1/2 cells reverted the suppressive effect of Suv39h1 on MyoD-induced target gene expression (Figure 4l). The same rescuing effect can also be observed on MyoD-responsive myogenic reporters, Myog-Luc and 4RE-Luc (Figure 4m). These results further underscored the functional importance of *Malat1* in modulating MyoD/Suv39h1 transcriptional activity.

### *Malat1* depletion accelerates skeletal muscle differentiation *in vivo*

**Figure 5:**
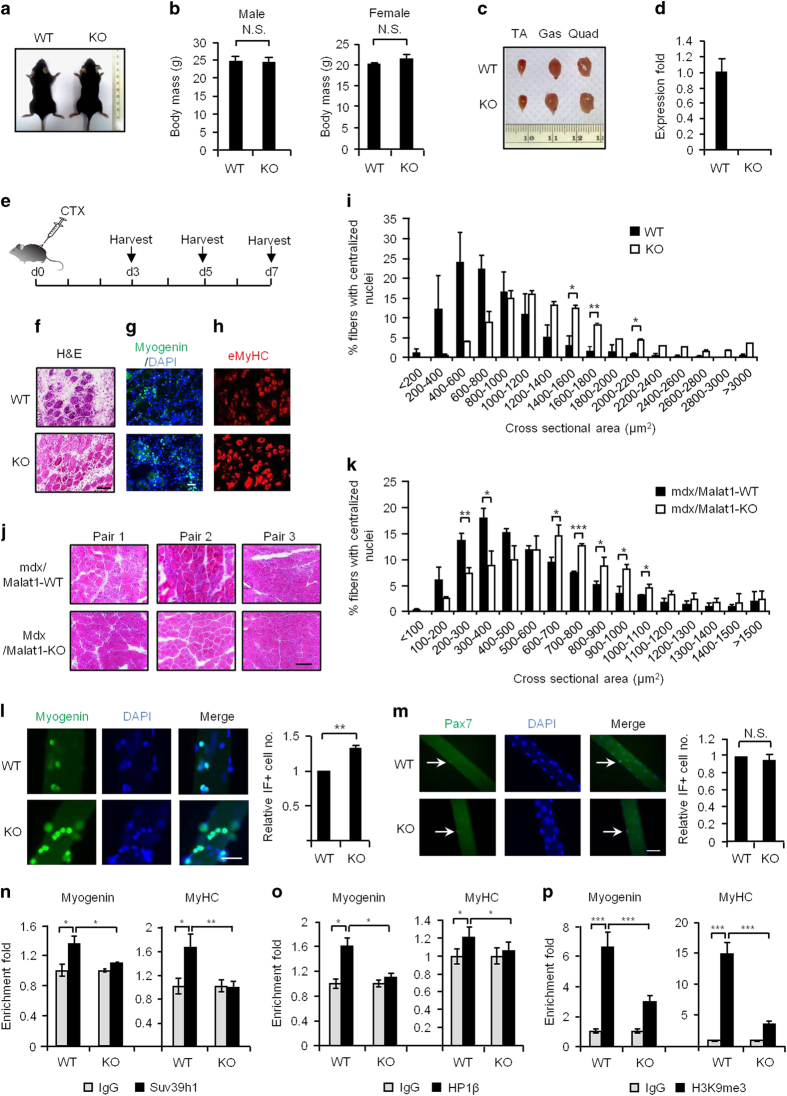
*Malat1* depletion leads to enhanced regeneration in acute or dystrophic injured mouse muscles. (**a**) Comparison of WT and *Malat1*-KO adult littermates showed no apparent difference. Representative images of one pair of mice were shown. (**b**) No difference was detected comparing the body mass of WT and KO male/female littermates or (**c**) comparing the size of three types of mouse limb muscles (Tibialis anterior, TA; Gastrocnemius, Gas; Quadriceps, Quad). (**d**) QRT-PCR detection of the *Malat1* expression in the TA muscles from WT or KO mice. (**e**) The scheme for CTX injection into *Malat1*-WT or KO mouse TA muscle and the tissue harvesting time points for subsequent analyses. (**f**) The above injected muscles were harvested on day 5 post injections and used for H&E staining. The images were taken at ×200 magnification. Scale bar=100 μm. (**g,** **h**) The above CTX-injected muscles were harvested on day 3 post injections, which were used for immunostaining of Myogenin or eMyHC, respectively. The images for Myogenin were taken at ×100 magnification. The images for eMyHC were taken at ×200 magnification. Scale bar=100 μm. (**i**) The cross-sectional area of the newly formed fibers on day 7 post-CTX injection with centralized nuclei was quantitated on the H&E staining sections using ImageJ (NIH, Bethesda, MD, USA). Three pairs of WT and KO mice were analyzed. Data are presented as mean±s.d. The *P*-value was calculated with Student’s *t*-test. (**j**) The TA muscles of mdx/*Malat1*-KO mice and littermate mdx/*Malat1*-WT mice were harvested at 5 weeks postnatal and used for H&E staining. Images from three pairs of representative mice are shown. Scale bar=100 μm. (**k**) The cross-sectional area of fibers with centralized nuclei was measured from the above H&E images. Three pairs of littermate mdx/*Malat1-*WT and mdx/*Malat1*-KO mice were analyzed. Data are presented as mean±s.d. The *P*-value was calculated with Student’s *t*-test. (**l**) Single myofibers were isolated from the extensor digitorum longus muscle of 2-month-old littermate WT and KO mice and cultured for 72 h before staining for Myogenin (green) and DAPI (blue). The number of Myogenin+ SCs was counted from three pair of littermates. Scale bar=50 μm. (**m**) The above myofibers were fixed immediately after the isolation and used for IF staining of Pax7 (green) and DAPI (blue). The number of Pax7^+^ satellite cells was counted from three littermates. Scale bar=100 μm. (**n**–**p**) ChIP-PCR analyses of Suv39h1, HP1β and H3K9me3 enrichment on the *Myogenin* or *MyHC* promoter *in vivo* using WT or KO TA muscle harvested 3 days post-CTX injection. The IP efficiency was calculated by setting IgG value as one. All PCR data were normalized to *GAPDH* mRNA and represent the average of three independent experiments±s.d. *P<0.05, **P<0.01, ***P<0.001.

Although no obvious developmental phenotype was observed in the recently generated *Malat1*-KO mice [38–40], we wanted to examine whether these mice would show any phenotype under certain circumstances, for example, the injury-induced muscle regeneration. In line with the previous report [40], the body size, weight and gross morphology of the KO mice displayed no obvious abnormality (Figure 5a and b). Further examination of their limb muscles did not reveal any evident difference from the WT littermates either, despite a sharp decrease in *Malat1* expression in KO muscles (Figure 5c and d). However, in the subsequent analyses of the cardiotoxin-induced muscle regeneration process (Figure 5e), a subtle but notable acceleration was observed in the KO mice compared with WT littermates by hematoxylin and eosin (H&E) staining of the muscle sections on day 7 post injury (Figure 5f). In the following immunostaining of the above sections, the increased number of cells positively stained for Myogenin and eMyHC also indicated enhanced muscle regeneration in *Malat1*-KO mice compared with WT littermates (Figure 5g and h). By measuring the cross-sectional areas (CSA) of the newly formed fibers with central nuclei, we found that the fiber size in KO mice was significantly larger compared with WT littermates (Figure 5i), confirming that the regeneration in KO mice was indeed accelerated. Interesting, heterozygous *Malat1* mice did not display such accelerated muscle regeneration (Supplementary Figure S5a–d), indicating a lack of haploinsufficiency. To further validate our findings, we also examined the effect of *Malat1* depletion in another regeneration model: the dystrophic muscle. We used the mdx mouse model of muscular dystrophy to generate mdx/*Malat1-*KO double-KO mice. Massive degeneration–regeneration was observed in the mdx mice of 3–6 weeks of age (Figure 5j). As expected, enhanced muscle regeneration was observed in the mdx/*Malat1*-KO mice compared with littermate mdx/*Malat1*-WT mice by H&E staining on cross-sections of TA muscle from 5-week-old mice (Figure 5j). By calculating the CSA of fibers with centrally localized nuclei we found an increased number of fibers with larger CSA in mdx/*Malat1*-KO mice than that in mdx/*Malat1*-WT mice (Figure 5k). To further test whether the above phenotype stemmed from the enhanced SC activities, we isolated single muscle fibers with SCs attached as an *ex vivo* model [30]. When induced to differentiate in the culture, SCs from the KO fibers displayed a higher myogenic competence as shown by increased Myogenin staining compared with the WT (Figure 5l, 1.3-fold increase of the positively stained cells). This is consistent with what we observed in C2C12 cells with *Malat1* knockdown (Figure 2), suggesting the increased myogenic capacity of the SCs contributed to the enhanced muscle regeneration in the KO mice. Interestingly, by staining for Pax7 on the freshly isolated fibers or on the cross-sections of TA muscles, we did not detect significant difference in the number of SCs in KO versus WT mice (Figure 5m and Supplementary Figure S5e), suggesting that *Malat1* depletion did not affect the number of quiescent SC pool. To further test whether the molecular mechanism uncovered from C2C12 myoblast applies *in vivo* in the SC cells, we performed *in vivo* ChIP for Suv39h1 or HP1β in the cardiotoxin-induced regenerating muscle from WT or KO mice. Reduced occupancy of Suv39h1 and HP1β at *Myogenin* and *MyHC* promoters was observed (Figure 5n and o); consistently, decreased level of H3K9me3 at these sites was also detected (Figure 5p and Supplementary Figure S6). Taken together, our data revealed that *Malat1* depletion in mice led to accelerated muscle regeneration through boosting the myogenic potential of SCs.

### Nuclear *Malat1* is directly targeted by miR-181a/Ago2 machinery upon myoblast differentiation

**Figure 6:**
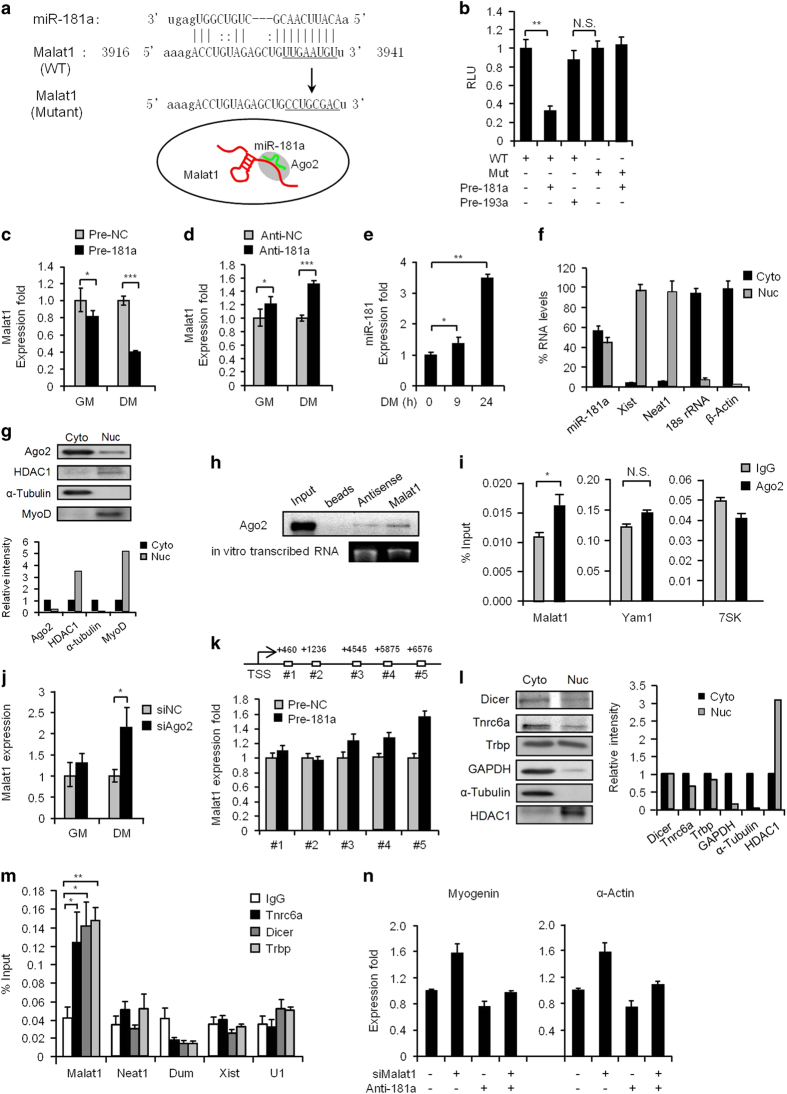
*Malat1* is directly targeted by miR-181a in the nucleus of myoblast through a Ago2-dependent nRISC machinery. (**a**) Upper: the predicted binding site between miR-181a and *Malat1-*WT sequence (5′-3916–3941-3′). A mutant was generated by mutating the seed sequence (UUGAAUGU, underlined) to CCUGCGAC. Lower: the hypothetical model depicting that *Malat1* transcript is directly targeted by miR-181a/Ago2 machinery in the myoblast nucleus. (**b**) The above-identified WT or mutated miR-181a-binding site was cloned into a pMIR luciferase reporter vector and transfected into the C2C12 cells with miR-181a (pre-181a) or miR-193a (pre-193a) precursor oligos. Luciferase activities were determined 48 h post transfection. (**c**) Pre-181a or negative control (Pre-NC) precursor oligos were transfected into C2C12 cells. The expression level of *Malat1* was examined by qRT-PCR after the cells were switched to DM or maintained in the growth medium (GM) for 48 h. (**d**) An antagomiR of miR-181a (Anti-181a) or a negative control (Anti-NC) oligo was transfected into C2C12 and the expression of *Malat1* was determined as above. (**e**) The expression level of miR-181a during C2C12 differentiation at 0, 9 and 24 h was determined by qRT-PCR. (**f**) The distribution of miR-181a in C2C12 cytoplasmic (Cyto) or nuclear (Nuc) fraction was determined by qRT-PCR. *Xist*, *Gapdh* and *U6* RNAs were used as controls to compare with. (**g**) Upper panel: cytoplasmic or nuclear level of Ago2 proteins was detected by western blotting. MyoD and HDAC1 were used as nuclear protein controls and α-Tubulin was used as cytoplasmic protein control. Lower panel: relative quantification of the band intensity was plotted. (**h**) RNA pull-down assay was performed with biotinylated *Malat1* FL transcripts to retrieve Ago2 in C2C12 nuclear lysates. Beads only or FL antisense transcripts were used as negative controls. The *in vitro* generated transcripts were visualized on the Agarose gel to show equal loading. (**i**) The C2C12 nuclear lysate was immunoprecipitated by an Ago2 antibody. The retrieved *Malat1* RNAs were detected by qRT-PCR. *Yam1* and *7SK* RNAs were also detected as controls. (**j**) Ago2 was knocked down by an siRNA oligo in C2C12 myoblast and the *Malat1* expression was examined by qRT-PCR after the cells were switched to DM or maintained in GM for 48 h. (**k**) Nuclear run-on assay was performed in C2C12 nuclei transfected with pre-181a or pre-NC oligos. Primers were designed to detect nascent transcription from five different locations of the *Malat1* gene. (**l**) Left: cytoplasmic or nuclear levels of Dicer, Tnrc6a and Trbp proteins were detected by western blotting. GAPDH and α-Tubulin were used as cytoplasmic protein control and HDAC1 was used as nuclear protein control. Right: relative quantification of the band intensity was also plotted. (**m**) The nuclear extract of C2C12 cells was immunoprecipitated by the indicated antibodies. The retrieved *Neat1*, *Dum*, *Xist*, *U1* or *Malat1* RNAs were detected by qRT-PCR. (**n**) C2C12 cells were transfected with the indicated oligos. Expression of the indicated myogenic genes was analyzed by qRT-PCR. All PCR data were normalized to *GAPDH* mRNA and represent the average of three independent experiments±s.d. All luciferase data were normalized to Renilla luciferase activities and represent the average of three independent experiments±s.d. *P<0.05, **P<0.01, ***P<0.001.

The interesting downregulation of *Malat1* at the very early stage of myogenic differentiation suggested that its expression must be tightly regulated. miRNA–lncRNA interaction is emerging as an important phenomenon. Although many studies favor the competing endogenous model, that is lncRNA acting as the sponges for miRNA to regulate its function, we thought about the alternative, that is, miRNA targeting *Malat1* for its downregulation because exploration of the Ago2 CLiP-seq (photo-crosslinking immunoprecipitation using Ago2 antibodies, followed by deep-sequencing of RNAs) in mouse embryonic stem cells [46] revealed a potential association of Ago2 protein with *Malat1* transcript (data not shown), suggesting that it could be bound and regulated by the miRNA/Ago2 silencing machinery (Figure 6a). We thus looked for miRNAs that could potentially target *Malat1* upon differentiation; this led to the discovery of a sequence complementarity between *Malat1* and 82 miRNAs (Supplementary Table S2) including miR-181a (Figure 6a), which happens to be a well-known regulator of skeletal myogenesis [47]. Indeed, by cloning the identified miR-18a binding region in *Malat1* into a luciferase reporter vector, we found that transfection of precursor oligos of miR-181a (pre-181a) but not a negative control or an irrelevant miR-193a downregulated the reporter activity (Figure 6b); moreover, mutating the predicted binding site (Figure 6a, Mutant) abolished this targeting effect (Figure 6b), suggesting that indeed miR-181a directly targeted *Malat1* through the above-identified site. In line with the finding, overexpression of miR-181a by transfecting the precursor miR-181a oligos led to a significant downregulation of the endogenous *Malat1* in differentiating C2C12 cells (Figure 6c, 60% decrease, Supplementary Figure S7a and b), whereas depletion of miR-181a by Antagomirs (Anti-181a) increased the expression of *Malat1* (Figure 6d, 1.5-fold, Supplementary Figure S7c). In contrast to the notable downregulation of *Malat1* expression in the early stage of C2C12 differentiation (Figure 1d), miR-181a expression level was found to gradually increase (Figure 6e), supporting their functional antagonization during this stage. Indeed, as previously reported [47], miR-181a is known for its pro-myogenic function in myoblast cells. Still, we were uncertain about whether this miR-181a/*Malat1* regulation could occur in the nucleus where *Malat1* was enriched, given that the miRNA-mediated silencing machinery is traditionally thought to mainly function in the cytoplasm. To eliminate our doubts, we examined the subcellular localization of miR-181a and detected a substantial amount of miR-181a in the nuclear portion (Figure 6f). Consistently, we also detected a high amount of Ago2 protein in C2C12 nuclei (Figure 6g), indicating that the miRNA/Ago2 machinery could possibly function in the nucleus of myoblast cells. To further demonstrate the involvement of Ago2 in this regulation, we showed that biotinylated FL *Malat1* transcripts could pull down an evident amount of Ago2 protein from nuclear lysates of early-differentiating C2C12 myoblasts (Figure 6h); moreover, antibodies against Ago2 also retrieved a significant level of endogenous *Malat1* transcripts from nuclear lysates (*Yam1* and *7SK* were not retrieved; Figure 6i), indicating an association between Ago2 protein and *Malat1* RNAs in the C2C12 nucleus upon differentiation. In keeping with this, knockdown of Ago2 markedly increased the expression of *Malat1* in differentiating myoblasts (Figure 6j). To further explore how miR-181/Ago2 downregulates *Malat1* transcripts, we first tested whether they could suppress *Malat1* transcription as increasing lines of evidence showed the non-canonical functions of nuclear miRNA/Ago in recruiting epigenetic silencing complex, which triggered transcriptional silencing [48]. However, by nuclear run-on assay, we did not detect a decrease in nascent *Malat1* transcription by miR-181a overexpression, suggesting that the regulation may not be at the transcriptional level; instead, miR-181/Ago2 may cause *Malat1* degradation through an nRISC machinery (Figure 6k). To test this notion, we found that, besides Ago2, multiple components of RISC including Dicer, Trbp, which were required for the recruitment of Ago2 to Dicer [49], and Tnrc6a/GW182, which navigated Ago2 to translocate into the nucleus [50], were detected in the nuclear portion of C2C12 cells (Figure 6l). Using RIP (Figure 6m) and RNA pull down (Supplementary Figure S7d) assays an association of these proteins with *Malat1* transcripts was also detected in the C2C12 nuclear lysates. The above findings supported the presence of nRISC in the C2C12 nucleus and its participation in degrading *Malat1* transcripts upon myoblast differentiation. Lastly, to confirm the functionality of the miR-181a/*Malat1* regulation, we further demonstrated that knockdown of *Malat1* could revert the anti-myogenic effect of anti-miR-181a treatment on myogenesis (Figure 6n). Altogether, the above data suggested that *Malat1* was reduced by miR-181a/Ago2 degradation through the nRISC-dependent machinery in the nucleus of myoblast cells upon early differentiation.

## Discussion

**Figure 7:**
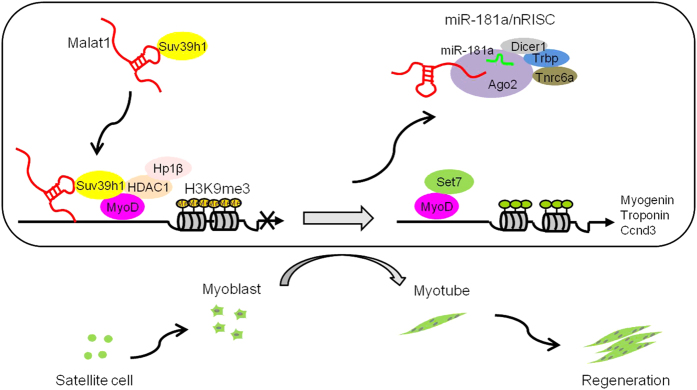
Schematic model of *Malat1* regulation of MyoD transcriptional activity during myogenesis. The model depicts the role of the miR-181a-*Malat1*-MyoD/Suv39h1 regulatory axis in myogenic differentiation and muscle regeneration. Upon injury, SCs are activated into committed myoblasts, which proliferate and differentiate into myotubes, leading to successful regeneration. In the proliferating myoblasts *Malat1* is present in high abundance and recruits Suv39h1 to MyoD-binding loci through tethering to the DNA loci; the subsequent formation and stabilization of the Suv39h1/HDAC1/HP1β complex leads to trimethylation of the Histone 3 lysine 9 (H3K9me3) and repression of the target gene expression. Upon differentiation, miR-181a expression is induced, causing *Malat1* degradation through their direct interaction and Ago2/nRISC-dependent machinery. The repressive complex is thus destabilized and replaced by Set7-containing activating complex, leading to monomethylation of Histone 3 lysine 4 (H3K4me1) and target gene activation. The myogenic differentiation represents a critical step during the entire muscle regeneration.

Our findings provided a comprehensive view of *Malat1* function in skeletal muscle cells and muscle regeneration, and also elucidated a novel role of *Malat1* in the epigenetic/transcriptional regulation of gene expression. As illustrated in Figure 7, upon injury, SCs are activated into committed myoblasts that proliferate and further differentiate into myotubes. In the proliferating myoblasts *Malat1* is present in high abundance and recruits Suv39h1 to MyoD-binding loci through its ability to tether with the MyoD target loci; subsequent formation and stabilization of the Suv39h1/HDAC1/HP1β complex leads to trimethylation of Histone 3 lysine 9 (H3K9me3) and repression of the target gene expression. At the onset of differentiation, miR-181a expression is induced, causing *Malat1* degradation through their direct interaction and Ago2-dependent nRISC machinery in the nucleus. The repressive complex is thus destabilized and replaced by Set7-containing activating complex, which allows MyoD trans-activation to occur on the target loci. The myogenic differentiation represents a critical step during the entire muscle regeneration, which is attributed to SCs.

*Malat1* is no doubt one of the first identified and one of the most well-studied lncRNAs. Despite numerous reports on its abundance in various cell types and overexpression in many human tumors, the exact cellular function of *Malat1* is still a matter under intense investigation. It is most popularly known for its regulation of RNA splicing through interacting with SR proteins, and is less known as an epigenetic modulator. However, Yang *et al.* [13] showed *Malat1* can bind to histone methyltransferases/demethylases such as SET2, although no further functional characterization was carried out. Our study is the first to show that *Malat1* interacts with Suv39h1, which is also a Set domain-containing methyltransferase, and provides detailed functional characterization of this interaction. Distinct from the gene-activating role reported in the above study, *Malat1* association with Suv39h1 leads to gene repression, underscoring its pleiotropic functions in gene regulation.

Our findings from using various protein/RNA-binding assays demonstrate a direct physical interaction between *Malat1* FL transcript and Suv39h1 protein (Figure 4). Knockdown of *Malat1* also hampered the association of the entire MyoD/Suv39h1/HP1β/HDCA1 complex with the target loci, suggesting that *Malat1* probably is important for the recruitment of Suv39h1 and stabilization of the complex on the target loci. In addition, by ChIRP assay (Figure 4f), we showed that *Malat1* is physically tethered to the recruitment site, that is, the promoter or enhancer region of the myogenic genes but exactly how the RNA/DNA interaction occurs needs further investigation. It is likely that the binding may depend on contact with some component of the transcription process and is not because of DNA sequence alone as CHART analysis has identified *MALAT1* enriching at active genes [51]. In addition, we also mapped the functional binding domain of *Malat1* with Suv39h1 through deletion mapping. Interestingly, results from both RNA pull-down and RNA/protein EMSA assays indicated that the 3′ domain (5 185–6 982) seems to be sufficient for efficient Suv39h1 binding. The 3′ end of *Malat1* is not produced by canonical cleavage/polyadenylation but instead by recognition and cleavage of a tRNA-like structure by RNAse P [52]. The processed *Malat1* transcript is protected from 3′ to 5′ exonucleases by highly conserved triple helical structures at the 3′ end, which promotes its RNA stability and high expression in most cell types [52]. Xu *et al.* [53] also identified the 3′ end of *Malat1* as an important motif in the invasion and metastasis of colorectal cancer. Nevertheless, the other domains (F1, F2 and F3) could also interact with Suv39h1 to various degrees, which is not surprising considering each fragment used in the mapping is large enough to form complex secondary structures. Interestingly, we also mapped the SET domains but not the chromodomain of Suv39h1 as the interacting domain with *Malat1*. This is consistent with the previous reports [54, 55] showing SET-containing proteins of the SET1 and SET2 families contain motifs in the pre-SET region or at the pre-SET/SET and SET/post-SET boundaries, which very tightly bind single-stranded DNA and RNA. Yang *et al.* [13], however, think it is the chromodomain of the Pc2 protein that binds with *Malat1* to function as modulators of Pc2 for ‘reading’ the histone code in HeLa cells. Further structural analysis will be needed to better understand the structure–function relationship.

Using myoblast differentiation as a model, our study mainly elucidated an inhibitory role of *Malat1* in the myogenic differentiation. However, *Malat1* may exert pluripotent functions in other aspects of SC activities. Of note, we observed a delay in myoblast proliferation upon *Malat1* knockdown, suggesting that it may also have a pro-proliferative function in myoblasts, which is in keeping with reports linking *Malat1* to cell cycle progression [56]. Moreover, it will be interesting to further explore the cellular mechanisms underlying these other functions in SCs. In addition, although *Malat1* expression is quickly downregulated at the early stage of differentiation, it sharply recovers when the myoblasts enter terminal differentiation (Figure 1d), suggesting that *Malat1* may have additional functions in promoting myotube fusion or maintaining myotube homeostasis. We must point out that in contradictory to our findings, Watts *et al. *[31] showed a slight decrease in *Myogenin* RNA after *Malat1* knockdown, leading to the conclusion that *Malat1* is a pro-myogenic factor. In a separate study, Han *et al.* [32] also reported reduced *Myogenin* mRNA expression in differentiating myotubes in response to partial *Malat1* inhibition. In both studies, the measurement was conducted 4 days into the differentiation whereas *Myogenin* induction usually occurs in the early phase. In addition, because of its anti-proliferative effect in the myoblast cells, it is necessary to adjust the cell confluence to comparable levels when inducing cell differentiation for the *in vitro* experiments, which could be another confounding factor for causing the discrepancy seen in our studies. Nevertheless, in addition to the multiple lines of evidence from using *in vitro* cell culture, we have also used knock-out mice model to strengthen our findings showing the anti-differentiation role of *Malat1*, which is consistent with a recent report that inhibition of *Malat1* could induce cancer cell differentiation [57]. We thus believe that our study provide substantial evidence to support the anti-myogenic action of *Malat1* during the early phase of differentiation. Still, *Malat1* is one of the multifunctional lncRNAs in skeletal muscle cells, and continuous exploration will bring many future surprises.

Given its known important functions in many cells, the high abundance in most cell types examined so far and its strong sequence conservation from human to zebrafish, it is surprising that the *Malat1*-KO mouse did not exhibit any obvious developmental phenotype. Our study is the first to report that *Malat1*-KO mouse indeed displays some phenotype in skeletal muscle tissues under stress (acute or chronic injury-induced regeneration), even though the skeletal muscle development appears normal. Although not dramatic, the accelerated regeneration was repeatedly seen in the KO mice, confirming that *Malat1* acted to repress the regeneration process. This can be attributed to some extent to its regulation of the SC myogenic ability as the SCs isolated from the KO mice displayed accelerated myogenic differentiation. However, we cannot rule out the possibility of reduced degeneration as well as contributions from other cell types in the injured muscle as regeneration is a process occurring in a complex niche environment and governed by intercalating signaling networks. In order to better elucidate the functional impact of its loss on SCs, it will be necessary to generate an SC-specific KO mouse model of *Malat1* in the future. It is also foreseeable that *Malat1*-KO mice may have other tissue- or condition-specific phenotypes that await to be discovered. Indeed, two recent reports published during the preparation of our manuscript demonstrated that *Malat1*-KO mice showed a delayed vessel extension in the retina [58] and decreased fertility [59] compared with WT littermates.

Although the high abundance and stability of *Malat1* could suggest a competing endogenous function titrating the amount of miR-181a, emerging reports start to demonstrate that the microRNAs binding could also reduce lncRNA stability [14]. Nonetheless, there is still limited mechanistic understanding for how miRNA-mediated targeting and degradation of lncRNAs occur mainly because canonically miRNA is believed to regulate mRNA expression through RISC machinery in the cytoplasm. However, in recent years several lines of evidence point to additional functions for miRNAs in the nucleus that have been largely ignored so far [48]. For example, the majority of cellular miRNAs are present in both the nucleus and the cytoplasm. Multiple components of the RISC machinery are present in the nuclear compartment. Our results clearly demonstrate that miRNAs can indeed target nucleus-residing lncRNAs for degradation through the nRISC machinery and highlights that the complexity of the non-coding RNA involved regulatory interactions.

Modulating the activities of transcription factors and epigenetic regulators on myogenic loci is crucial for skeletal muscle differentiation. Our findings underscore the important role of *Malat1* in this process. It is one of the few lncRNAs identified so far that have important functions in myogenesis; we nevertheless believe that many such lncRNAs exist and will need to be characterized in the near future. Together with transcription factors and chromatin regulators, they form complex regulatory networks orchestrating the precise gene transcription. Better knowledge of their acting mechanisms will enable the understanding and modulation of muscle regeneration in pathophysiological conditions.

## Materials and methods

### Cell culture

Mouse C2C12 myoblast cells (CRL-1772) and C3H/10T1/2 (10T1/2) fibroblast cells (CCL-226) were obtained from ATCC and cultured in DMEM medium (12800-017, Gibco) with 10% fetal bovine serum (10270-106, Gibco), 100 units/ml of penicillin and 100 μg of streptomycin (15140-122, Gibco) (growth medium, or GM) at 37 °C in 5% CO2. To induce cell differentiation, near confluent cells were switched to DMEM containing 2% horse serum (16050-122, Thermo Fisher Scientific) (differentiation medium, or DM). Muscle satellite cells (SCs) of 2 months old Pax7-nGFP mouse [60] were isolated as previously reported [61]. The Pax7 positive cells were sorted based on the GFP signals. Sorted-out SCs were collected upon sorting as quiescent SCs or cultured in F10 medium (F0723, Merck Millipore) with 10% horse serum (26050088, Invitrogen, Carlsbad, CA, USA).

### Cell proliferation assay

MTT assays were conducted following the instructions of manufacturer (M6494, Invitrogen). Culture volume (1/10) of MTT solution (5 mg ml^−1^) was added to cultured cells incubated in a CO_2_ incubator at 37 °C for 4 h. After the incubation medium is removed and DMSO was added to dissolve the crystals with pipetting up and down. A volume of 100 μl of DMSO solution was transferred to a 96-well plate, and the absorbance was measured at 550 nm with background subtraction at 650 nm. BrdU assay was conducted as the manufacturer’s instructions (B5002, Sigma-Aldrich). Growing cells on coverslips were incubated in culture medium with 10 μM BrdU for 20 min. Cells were then washed for three times in phosphate-buffered saline (PBS) and fixed with fixative solutions (3 volumes of glycine solution (50 mM, pH 2.0) with 7 volumes of pure ethanol) for 45 min at room temperature (RT). Cells were washed in PBS twice and then incubated in 4 M HCl for 10–20 min at RT to denature DNA. Cells were washed for three times to neutralize the pH and then incubated in the incubation buffer (PBS containing 0.5% bovine serum albumin and 0.1% Tween 20) to block unspecific binding. Anti-BrdU antibody was then added into the incubation buffer; after 1 h incubation at RT, cells were washed in PBS. The coverslips were then mounted with antifade mounting solution with 4,6-diamidino-2-phenylindole (DAPI; P36931, Invitrogen). Pictures were captured with a fluorescence microscope (Zeiss, Jena, Germany).

### Cell fractination

RNA from different subcellular fractions of C2C12 cells were isolated as previously described [62]. Cell pellet was lysed with 175 μl per 10^6^ cells of cold RLN1 solution (50 mM Tris HCl pH 8.0; 140 mM NaCl; 1.5 mM MgCl_2_; 0.5% NP-40; 2 mM Vanadyl Ribonucleoside Complex) and incubated 5 min on ice followed by centrifuge at 300 g and 4 °C for 2 min. The supernatant was saved as cytoplasmic fraction. The pellet was resuspended in 175 μl/10^6^ cells of cold RLN2 solution (50 mM Tris HCl pH 8.0; 500 mM NaCl; 1.5 mM MgCl2; 0.5% NP-40; 2 mM Vanadyl Ribonucleoside Complex) and incubated on ice for 5 min. The suspension was then centrifuged at 4 °C and 16360 g for 2 min and the supernatant was saved as nuclear-soluble fraction and the pellet was kept as the nuclear-insoluble fraction. RNAs from different fractions were isolated by Trizol (15596018, Invitrogen).

### Single myofiber isolation

Single myofibers were isolated as previous described [61]. Briefly, the extensor digitorum longus muscles were carefully cut from tendon to tendon and digested in F10 medium containing Collagenase type II (800 U ml^−1^) at 37 °C in a water bath for 75 min. After digestion, large bore pasteur pipette was used to flush the muscles in warm medium to release myofibers. Live single myofibers were transferred to a new dish and the operation was repeated for two to three times for removing dead fibers and debris. Isolated single myofibers were further cultured in F10 medium with 10% horse serum.

### Plasmids

The mouse *Malat1* FL expression plasmid was obtained from Dr Kannanganattu V Prasanth [11]. The mouse *Malat1* FL plasmid used for *in vitro* transcription was obtained from Dr Shinichi Nakagawa. Gal4-MyoD and Gal4 luciferase reporter plasmids were obtained from Dr Zhenguo Wu. The Suv39h1 expression plasmid was obtained from Dr Asoke K Mal [35]. The plasmids expressing full length or fragments of Suv39h1-GST, or GST only in bacteria were obtained from Dr Weiguo Zhu [63]. Myogenin-Luc, MCK-Luc, MyHC-Luc and Tnni2-Luc were used as described [26, 64]. Renilla luciferase reporter plasmid was obtained from Promega and used according to the manufacturer’s instructions (E1960, Promega, Madison, WI, USA). To construct *Malat1* luciferase reporter plasmids, a 45 bp fragment encompassing WT mmu-miR-181a-5p-binding site (5′-cAaagacctgtagagctgttgaatgtttgcagctggca-3′) or mutated binding site (5′-cAaagacctgtagagctgcctgcgacttgcagctggca-3′) was cloned into pMIR-report vector (AM5795, Life Technologies, Carlsbad, CA, USA) between Spe| and Sac| sites. To construct *Malat1* shRNAi viral vector, a 61 bp fragment (5′-gATCCggTgAATgAgTgATAAgTAATTCAAgAgATTACTTATCACTCATTCACCTTTTTTg-3′) harboring the target sequences was cloned into pSIREN RetroQ vector (631526, Clontech, Mountain View, CA, USA).

### Transfection and infection

Transient transfection of siRNA oligos or plasmids was conducted with Lipofectamine 2000 following the instructions from the manufacturer (11668-019, Invitrogen). For the transfection of *in vitro*-transcribed RNA, RNA was heated at 70 °C for 10 min. Lipofectamine 2000 was added directly to the RNA and then mixed by vortex. DMEM medium without serum or antibiotics was then added to the mixture and mixed by vortex. The final mixture was added to cells dropwise. For dual luciferase reporter assay, the firefly luciferase reporter plasmids were transfected to cells together with Renilla luciferase reporter plasmid, which worked as transfection efficiency control. To generate C2C12 cells with stable knocking down of *Malat1*, empty pSIREN-RetroQ retroviral vector of pSIREN/sh*Malat1* along with the packaging plasmid were transfected into HEK293T cells. Forty-eight hours after transfection, culture supernatants were collected and used for infecting C2C12 cells, which were later selected by 5 μg ml^−1^ puromycin.

### Oligonucleotides

Precursor miRNA oligos were obtained from Life Technologies. siRNA oligos against mouse *Malat1* and Ago2 were obtained from Ribobio Technologies (Guangzhou, China). Anti-miR-NC and miR-181a were obtained from Ribobio and transfected at a final concentration of 100 nM. siAgo2 oligos or miRNA oligos (50 nM) were used for transient transfections. *Malat1* siRNAs were transfected at a final concentration of 150 nM. The sequences of oligonucleotides used for siRNA knockdown, RT-PCR and ChIP-PCR were provided in the Supplementary Table S3.

### Quantitative real-time PCR

Total RNAs were extracted using TRIzol reagent (Invitrogen). Expression of mRNA was performed with SYBR Green Master Mix (4368708, Applied Biosystem, Foster City, CA, USA) as previously described using GAPDH for normalization [26, 64]. Expression of mature miRNAs was determined using the miRNA-specific TaqMan microRNA assay kit (Applied Biosystem) on an ABI PRISM 7900HT Sequence Detection System (Applied Biosystem). U6 was used for normalization.

### Northern blotting assay

An amount 10 μg of total RNAs were loaded in each well of 1.2% formaldehyde-denaturing agarose gel and after electrophoresis transferred to a charged nylon membrane overnight. After the transfer RNA was crosslinked using a UV crosslinker (Stratagene, San Diego, CA, USA) and pre-hybridized with ULTRAhyb hybridization buffer (AM8670, Applied Biosystems). Probes against mouse *Malat1* transcript were amplified with the following PCR primers (forward 5′-GTTACCAGCCCAAACCTCAA-3′; reverse 5′-CTACATTCCCACCCAGCACT-3′) and labeled with [α-32P]dCTP with RadPrime DNA labeling system (Invitrogen). Probes were boiled and hybridized with membrane overnight at 42 °C. After hybridization, the membrane was washed twice with 2× SSC buffer and 0.1% SDS at room temperature and once with 0.1× SSC and 0.1% SDS at 55 °C for 30 min.

### Fluorescence *in situ* hybridization

*In situ* hybridization was performed following the protocol from Dr KV Prasanth [11]. Probes were first amplified with PCR using *Malat1* expression plasmid as template. PCR products were then purified, nick-translated and labeled with Green d-UTP (Abbott, Chicago, IL, USA) using a nick translation kit (Abbott). Cells were fixed with 2–4% freshly prepared paraformaldehyde (in 1× PBS) pH 7.2 for 15 min at RT. The cells were permeabilized with 0.2–0.5% Triton X-100, 2 mM VRC (NEBiolabs, Ipswich, MA, USA) on ice for 5–10 min and then washed two times in 2× SSC for 10 min. In all, 200 μg or more of the probe and yeast tRNA (Sigma; 20 μg) were lyophilized and redissolved in 10 μl deionized formamide (Ambion, Carlsbad, CA, USA), and denatured at 75–100 °C for 10 min and immediately chilled in ice for 3–5 min. In all, 10 μl of hybridization buffer was added to each reaction to make a final hybridization cocktail of 20 μl per coverslip. Coverslips were incubated at 37 °C overnight in a humidified chamber. After the hybridization, coverslips were washed in 2× SSC, 50% formamide (pH 7.2) for 3×5 min at 42 °C, 2×SSC (pH 7.2) for 3×5 min at 42 °C, 1× SSC (pH 7.2) for 3×5 min at 42 °C and 4×SSC for 2×10 min at RT. Coverslips were mounted with ProLong Gold Antifade Reagent with DAPI (Invitrogen). Pictures were captured in Kahlman frame, giving an average of two scans using the Olympus microscope FV1000 and the accompanying software FV10-ASW (version 01.07.02.02, Olympus, Tokyo, Japan).

### RNA immunoprecipitation

RIP assay was conducted as previously described [65]. Two micrograms of antibodies against Suv39h1 (Millipore, Darmstadt, USA), Ago2 (Abcam, Cambridge, MA, USA), Dicer (Santa Cruz Biotechnology, Santa Cruz, CA, USA), Trbp (Abcam), Tnrc6a (Santa Cruz Biotechnology) or isotype IgG (Santa Cruz Biotechnology) were used in this assay. Pulled down RNA were resuspended in 20 μl of RNase-free water, and cDNAs were obtained from reverse transcription. qRT-PCR was performed with cDNA by using SYBR Green Master Mix (Applied Biosystems). Relative enrichment is calculated as the amount of amplified DNA normalized to values obtained after normal IgG immunoprecipitation.

### RNA-seq and data analysis

For library construction, we used a protocol as described before [66]. The purified library products were evaluated using a Bioanalyzer (Agilent) and SYBR qPCR and sequenced on an Illumina Hi-seq 2000 sequencer (pair-end with 50 bp). Sequenced fragments were mapped to reference mouse genome using TopHat. Cufflinks was then used to estimate transcript abundances of RNA-Seq experiments. Abundances were reported in Fragments Per Kilobase per Million, which is conceptually analogous to the reads per kilobase per million reads mapped used for single-end RNA-seq. Differentially expressed genes were identified using Cuffdiff with default parameters. A false discovery rate-adjusted *P*-value (or *q*-value) of 0.05 was used as the cutoff in the analysis. [67].

### Immunoblotting, immunostaining and immunohistochemistry

For western blotting, total cell proteins were prepared and used as previously described [64]. The following dilutions were used for each antibody: Myogenin (Santa Cruz Biotechnology; 1:2 000), YY1 (Santa Cruz Biotechnology; 1:2 000), Troponin (Sigma; 1:2 000), MyHC (Sigma; 1:2 000), α-Actin (Sigma; 1:2 000), α-Tubulin (Sigma; 1:5 000), MyoD (Santa Cruz Biotechnology; 1:2 000), Suv39h1 (Millipore, 1:2 000), HDAC1 (Abcam, 1:2 000), HP1β (Abcam, 1:2 000), Ago2 (Abcam, 1:2 000), Dicer (Santa Cruz Biotechnology, 1:2 000), Trbp (Abcam, 1:5 000), Tnrc6a (Santa Cruz Biotechnology, 1:2 000) and GAPDH (Santa Cruz Biotechnology; 1:5 000). Immunofluorescence on cultured cells was performed using the following antibodies: MyHC (Sigma; 1:350), Myogenin (Santa Cruz Biotechnology, 1:400) and Srsf2 (Abcam; 1:800). Immunofluorescence staining on frozen muscle sections was performed using the following antibodies: Laminin (Santa Cruz Biotechnology, 1:400), Myogenin (Santa Cruz Biotechnology; 1:400) and eMyHC (Leica, 1:300). Immunofluorescence on single myofibers was performed using the following antibodies: Pax7 (Developmental Studies Hybridoma Bank; 1:200) and Myogenin (Santa Cruz Biotechnology; 1:200). Hematoxylin and eosin (H&E) staining on frozen muscle sections was performed as previously described [68]. All fluorescent images were captured with a fluorescence microscope (Leica).

### Chromatin immunoprecipitation

ChIP assay was performed as previously described [29]. Five micrograms of antibodies against Suv39h1 (Millipore), Hp1β (Abcam), HDAC1 (Abcam), trimethyl-histone H3-K9 (Abcam), MyoD (Santa Cruz Biotechnology), Set7 (Abcam) or isotype IgG (Santa Cruz Biotechnology), which was applied as a negative control, were used in the assay. Pulled down DNAs were resuspended in 20 μl of water. qRT-PCR was performed with 1 μl of immunoprecipitated material by using SYBR Green Master Mix (Applied Biosystems). Relative enrichment is calculated as the amount of amplified DNA normalized to input and relative to values obtained after normal IgG immunoprecipitation.

### Chromatin isolation by RNA purification

ChIRP was conducted as previously described [45]. Biotin-labeled antisense oligos (20  nucleotides long) were designed and divided into odd and even pools. Briefly, C2C12 cells were harvested and crosslinked with 1% glutaraldehyde. An amount of 100 mg of cell pellet were used for one pull down. Cells were sonicated at 4 °C for 3 h to shear the DNA to 100–500 bp. Pooled odd and even probes were hybridized with sonicated chromatins and then pulled down by C-1 streptavidin beads (65001, Invitrogen). Pulled down RNA and DNA were isolated. Isolated RNA was reverse-transcribed using both oligodT and random hexamer, and the cDNA was used to perform qRT-PCR to check the pull-down efficiency. qRT-PCR was conducted with retrieved DNA using the primers for ChIP to check the enrichment of *Malat1* at certain DNA loci.

### RNA EMSA

EMSA for RNA was performed as previously described [69]. GST alone or GST-fused proteins were expressed in DE3 bacteria and induced by IPTG for 4 h at 28 °C and purified by glutathione-Sepharose 4B beads (GE Healthcare, Waukesha, WI, USA). *In vitro*-transcribed RNAs were heated to 90 °C for 2 min with 10× RNA structure buffer (100 mM Tris pH 7, 1 M KCl, 100 mM MgCl_2_) and then put on ice for 2 min followed by incubation at RT for 20 min. Reaction mixtures containing 50 mM Tris HCl pH 7.0, 150 mM NaCl, 0.25 mg ml^−1^ tRNA, 0.25 mg ml^−1^ bovine serum albumin, RNA and protein were mixed and incubated at 37 °C for 10 min. Following incubation, mixtures were immediately loaded to 0.8% non-denaturing agarose gels with loading buffer in 0.5× TAE.

### Animal studies

*Malat1^−/−^* KO mice were obtained from Dr Shinichi Nakagawa, and the genotyping of the mice was conducted following the procedures previously described in their paper [40]. C57 BL/10 ScSn-Dmd^mdx^ mice were obtained from the Jackson Laboratory (Bar Harbor, ME, USA). *Malat1* ^−/−^ male mice were crossed with homozygous Dmd^mdx/mdx^ female mice to generate *Malat1* ^+/^^−^; Dmd^mdx/y^ male mice. *Malat1* ^+/−^; Dmd^mdx/y^ male mice were then bred with Dmd^mdx/mdx^ female mice to obtain *Malat1* ^+/−^; Dmd^mdx/mdx^ female mice. *Malat1* ^+/−^; Dmd^mdx/y^ male mice and *Malat1* ^+/−^; Dmd^mdx/mdx^ female mice were then crossed to generate *Malat1* ^−/−^; Dmd^mdx/y^ and *Malat1* ^−/−^; Dmd^mdx/mdx^ mice, which were referred as mdx/*Malat1*-KO mice. Littermate mdx/*Malat1-*WT mice were used as controls. Mice were housed in the animal facilities of The Chinese University of Hong Kong (CUHK) under conventional conditions with constant temperature and humidity, and were fed a standard diet. Animal experimentation was approved by the CUHK Animal Experimentation Ethics Committee (Ref No. 14-059-GRF). Postnatal muscles were obtained from C57/BL6 mice for RNA extraction followed by RT-PCR analysis. For Cardiotoxin studies, ~8-week-old mice were injected with 50 μl of 10 μg ml^−1^ Cardiotoxin (Latoxan, France) solution into each tibialis anterior muscle. Muscles were harvested at designated time points for further analysis.

### Statistical test

The statistical significance was assessed by the Student’s *t*-test. *P<0.05, **P<0.01 and ***P<0.001.
