# Supplementary material for: Malat1 regulates myogenic differentiation and muscle regeneration through modulating MyoD transcriptional activity
Source: Cell Discov. 2017 Mar 14;3:17002–. doi: 10.1038/celldisc.2017.2 (PMC5348715; doi:10.1038/celldisc.2017.2)
Supplement: Supplementary Information [file celldisc20172-s1.pdf]

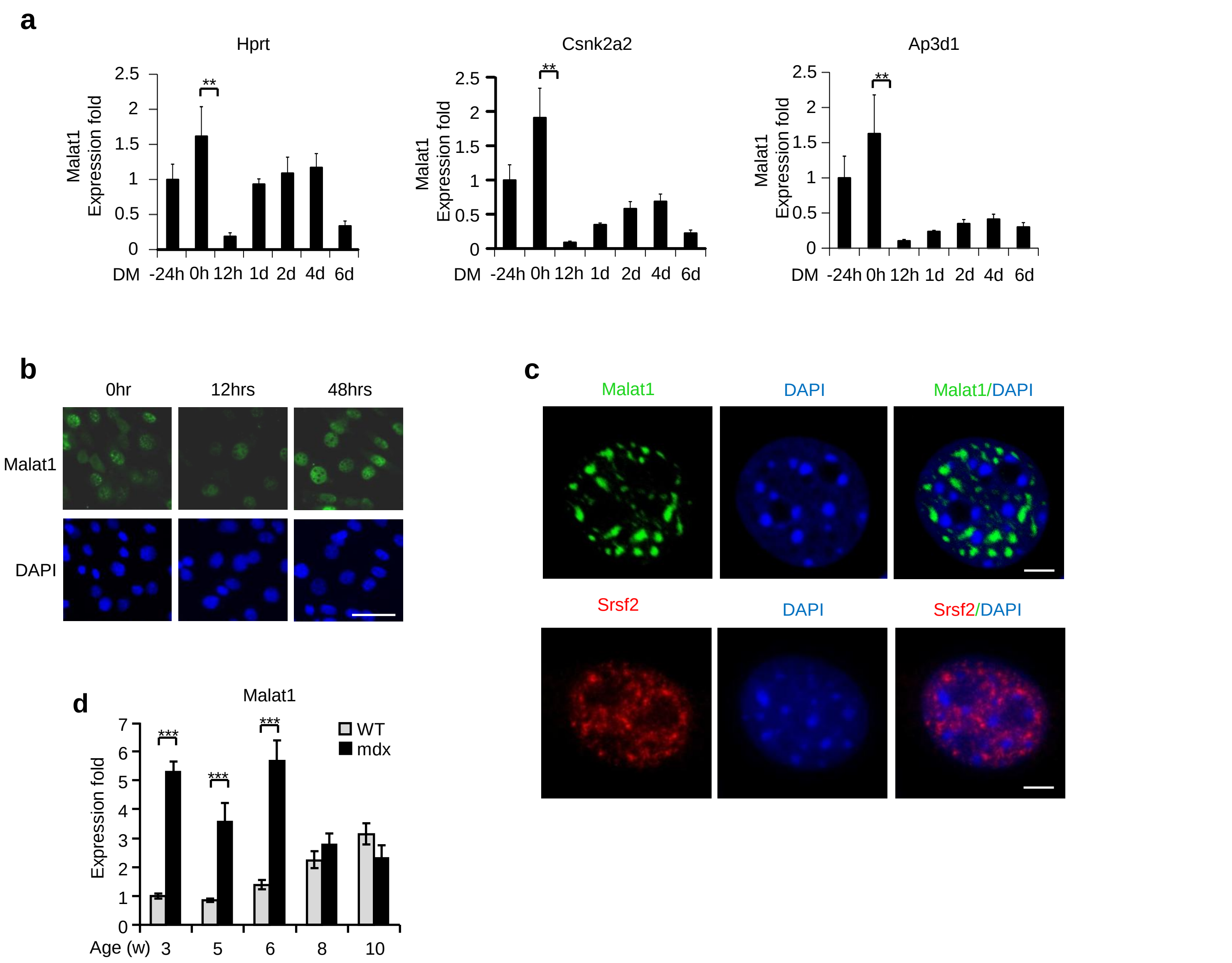

Supplementary Figure S1, Related to Figure 1. Expression dynamics of *Malat1* during C2C12 differentiation. (a) *Malat1* expression was measured by RT-qPCR using various normalization controls, Hprt, Csnk2a2 and Ap3d1. (b) *Malat1* is expressed in nucleus of both C2C12 myoblasts and myotubes during the myogenic differentiation. A down-regulation at 12hr and recovery at 48hr was observed. Scale bar = 50 µm. (c) *Malat1* is located in the nucleus of C2C12 cell and shows a speckle distribution pattern. Srsf2 was also stained as a nuclear speckle marker. Scale bar = 5 µm. (d) The expression of *Malat1* in muscles from WT or dystrophic mdx mice at the indicated age.

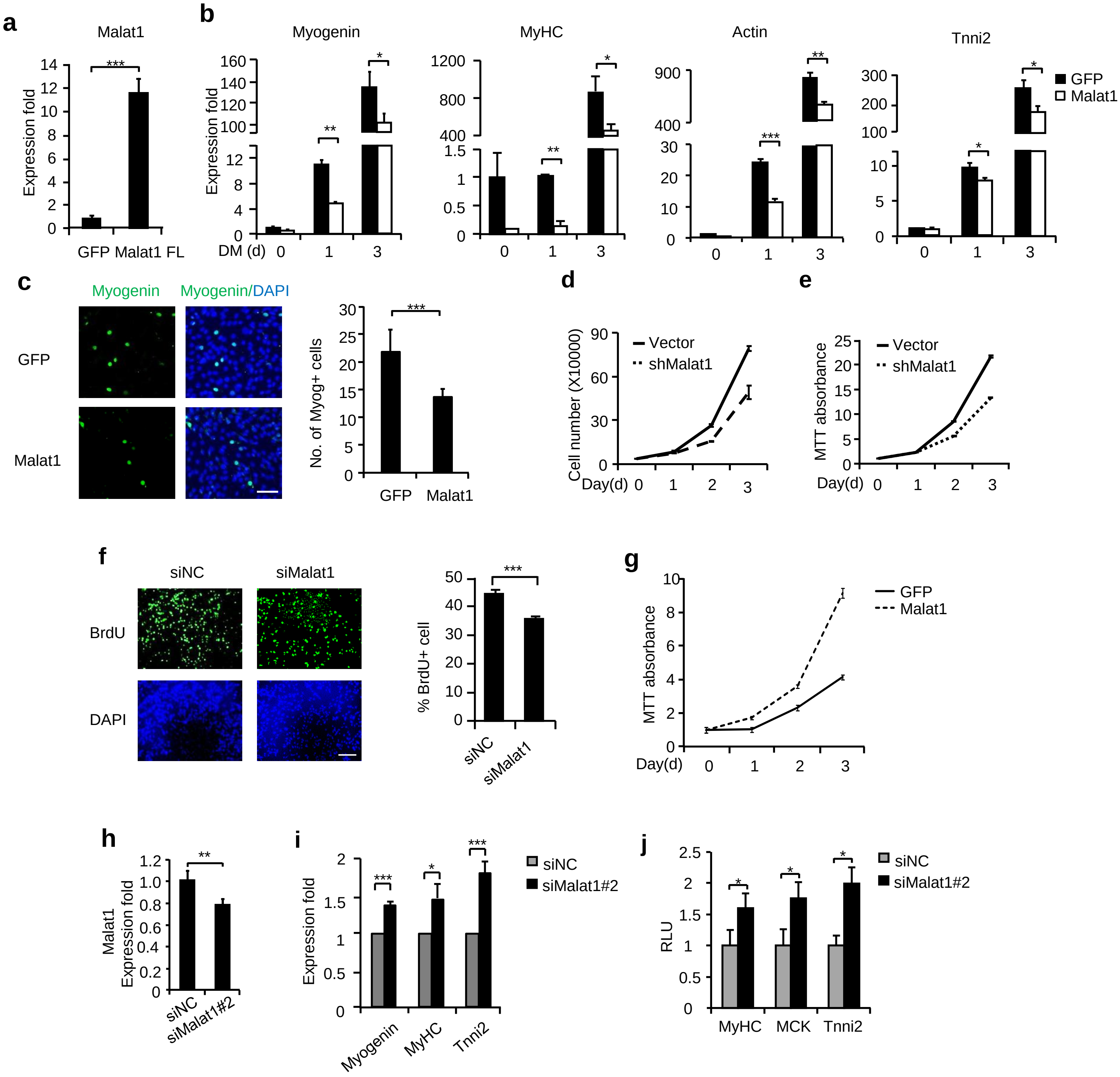

Supplementary Figure S2, Related to Figure 2. ***Malat1* inhibits myogenic differentiation and accelerates cell proliferation**. (a) *Malat1* expression was increased by transfecting C2C12 cells with 25nM *in vitro* transcribed *Malat1* transcripts as compared to negative control transfected with GFP transcripts. The above *Malat1* transfected cells displayed delayed differentiation as shown by (b) the down-regulation of various markers including Myogenin, MyHC, Actin and Tnni2 mRNAs and (c) the decreased number of Myogenin expressing cells by IF staining. The cells stably expressing *shMalat1* displayed a delayed proliferation rate as assessed by (d) viable cell counting assay or (e) MTT assay during a proliferating course of 3 days. (f) C2C12 cells were transfected with the above siRNA oligos against Malat1 and stained with BrdU. The positively stained cells were counted from at least 10 fields. (g) Overexpression of *Malat1* accelerated C2C12 proliferation as assessed by viable cell counting assay during a course of 3 days. (h-j) A second siRNA oligo (#2) was used to knockdown *Malat1* and the pro-myogenic effect was observed as assessed by expression of myogenic markers and myogenic reporter activities. RLU, relative luciferase unit. PCR data were normalized to GAPDH mRNA and represent the average of three independent experiments ± S.D. Luciferase data were normalized to Renilla luciferase activities and represent the average of three independent experiments ± S.D. *p<0.05, ** p<0.01, ***p<0.001.

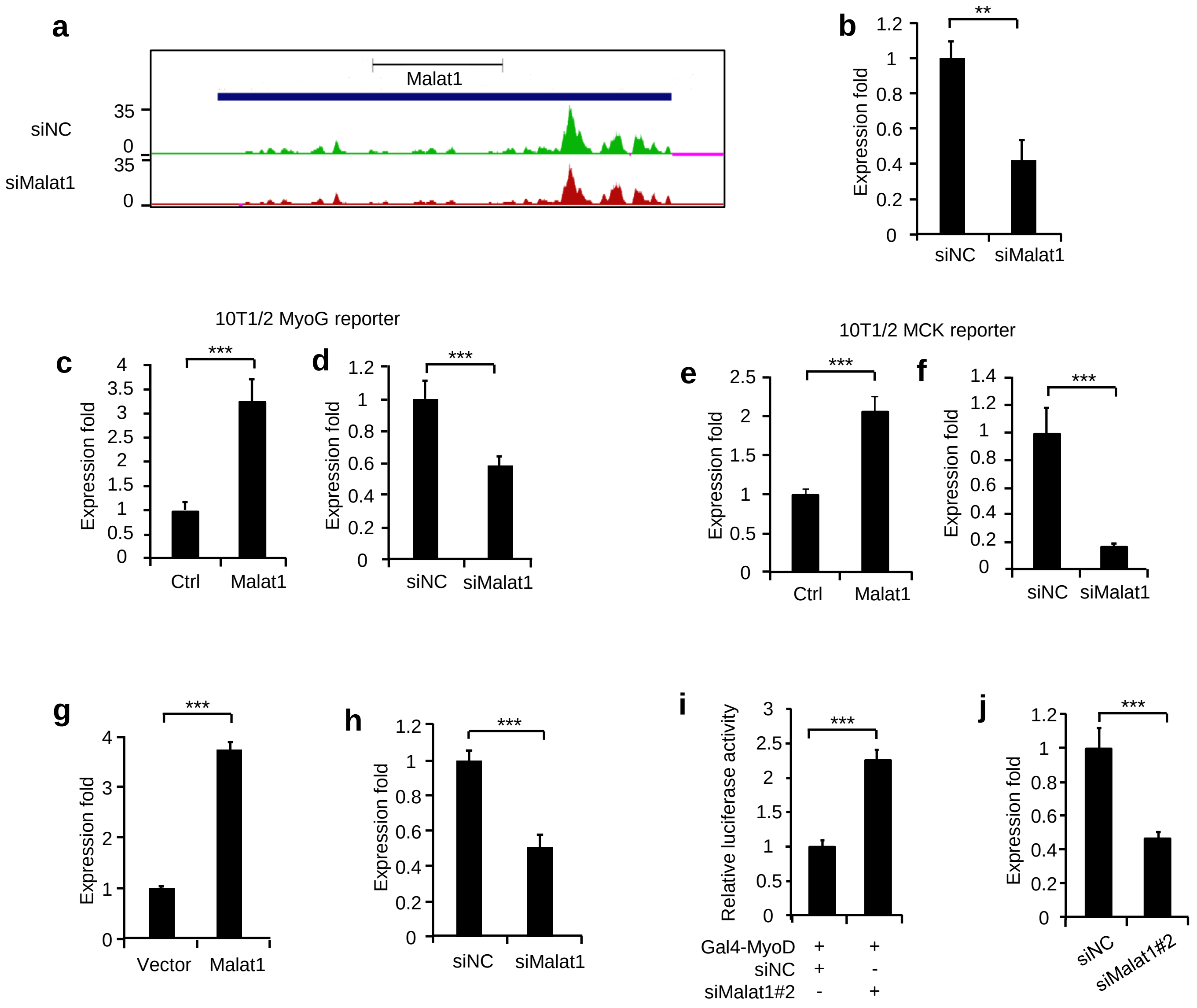

Supplementary Figure S3, Related to Figure 3. *Malat1* level in overexpression or knockdown cells. (a) Genomic snapshot from RNA-seq showing the expression of *Malat1* in C2C12 cells treated with siNC or si*Malat1* oligos. (b) RT-qPCR confirmed the knockdown of Malat1 by siRNA oligos in the samples used for RNA-Seq. PCR data was normalized to GAPDH mRNA. (c) The overexpression level of *Malat1* in 10T1/2 cells in the Myogenin luciferase reporter assay in Figure 3F was confirmed by RT-qPCR. (d) The knockdown of *Malat1* by siRNA oligos in 10T1/2 cells in the Myogenin luciferase reporter assay in Figure 3G was confirmed by RT-qPCR. (e) The overexpression level of *Malat1* in 10T1/2 cells in the MCK luciferase reporter assay in Figure 3F was confirmed by RT-qPCR. (f) The knockdown of *Malat1* by siRNA oligos in 10T1/2 cells in the MCK luciferase reporter assay in Figure 3G was confirmed by RT-qPCR. (g) The overexpression level of *Malat1* in 10T1/2 cells in Figure 3J was confirmed by RT-qPCR. (h) The knockdown of *Malat1* by siRNA oligos in 10T1/2 cells in Figure 3K was confirmed by RT-qPCR. (i) 10T1/2 cells were co-transfected with the Gal4 luciferase reporter, si*Malat1*#2 or siNC oligos and the Gal4-MyoD vector. Luciferase activities were measured after differentiating the cells for 48hrs. (j) The knockdown of *Malat1* by si*Malat1*#2 oligos in 10T1/2 cells in the above transfection is confirmed by RT-qPCR. PCR data were normalized to GAPDH mRNA and represent the average of three independent experiments ± S.D.

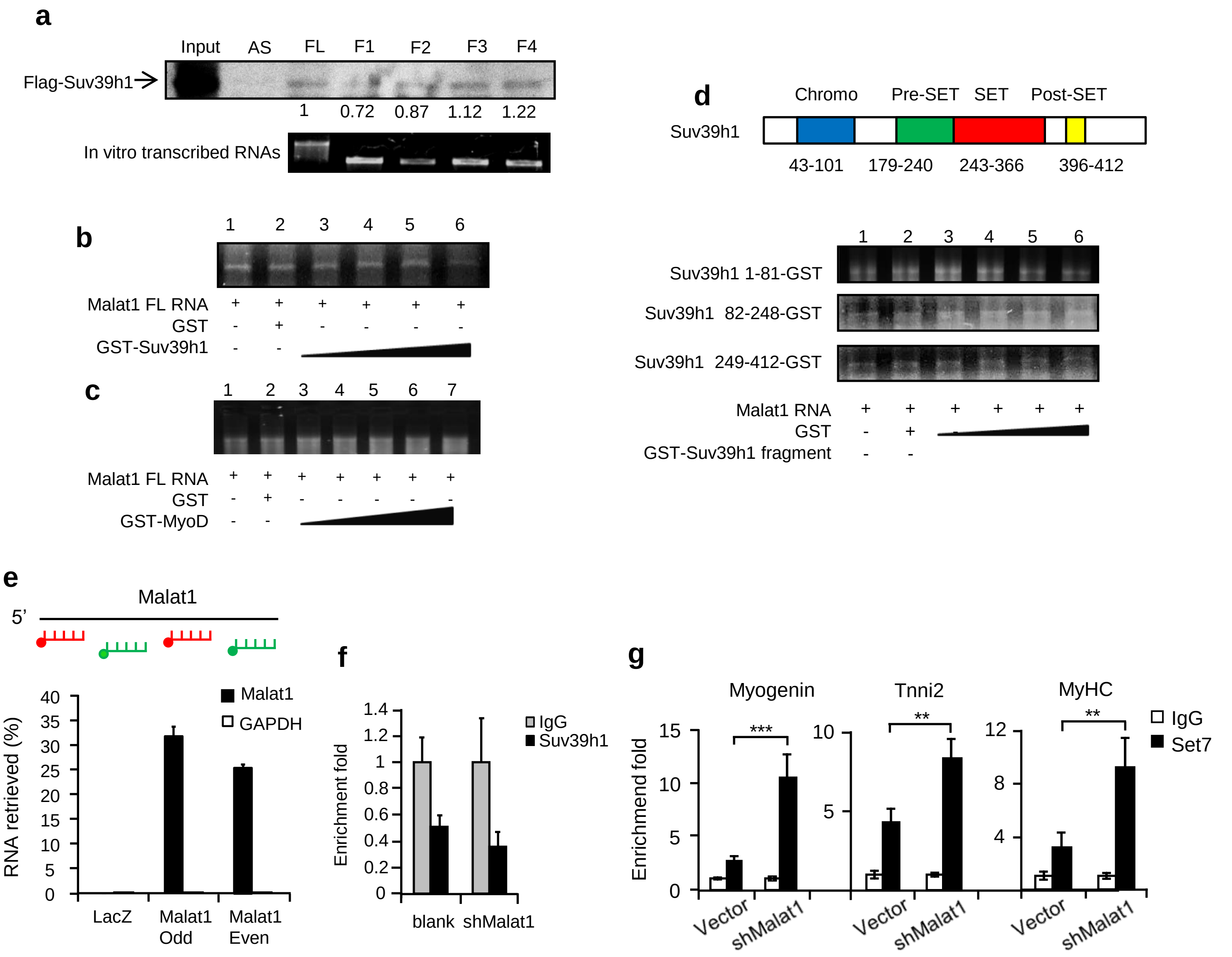

Supplementary Figure S4, Related to Figure 4. *Malat1* interacts with Suv39h1 to regulate MyoD trans-activation. (a) C2C12 cells overexpressing Flag-tagged Suv39h1 was used in RNA pull down assay. The retrieved protein was detected by antibody against Flag. The result demonstrated an association between Flag-tagged Suv39h1 with full length (FL) *Malat1* transcripts; the association with fragment F2, F3 and F4 was also evident but relatively weak with F1 fragment. (b) Electrophoretic Mobility Shift Assay (EMSA) detected an association between purified GST-Suv39h1 protein and in vitro transcribed full length *Malat1* (repeated for Figure 4e). A gradual shift was observed as the concentration of GST-Suv39h1 increases (lane 3 to 6); 1600 nM yielded an evident shift (lane 6); GST protein alone did not cause the shift (lane 2). (c) Such association was not detected when purified GST-MyoD protein was used in the EMSA with *Malat1* FL RNA. (d) EMSA detected a shift of *Malat1* by GST-Suv39h1 249-412 fragment (containing Set and Post-Set domain); no shift by GST-Suv39h1 82-248 fragment (Pre-Set containing) or GST-Suv39h1 1-181 (Chrome domain) was observed. (e) ChIRP assay to detect the association of *Malat1* with the MyoD target loci. Top: Schematic illustration of design of antisense DNA tiling probes grouped into "even" (green) and "odd" (red) sets based on their positions along the *Malat1* RNA. Bottom: Both even and odd tiling oligos effectively retrieved *Malat1* but not *GAPDH* RNAs from chromatin. As negative control, LacZ tiling oligos retrieved very low amount of *Malat1* RNAs. (f) A negative control region which has no MyoD or Suv39h1 binding shows no enrichment for Suv39h1 immunoprecipitation. (g) Knock-down of *Malat1* by the shRNA led to the increased enrichment of Set7 on the promoter of Myogenin, Tnni2 and MyHC. The data were represented as fold change compared to the level of IgG pull-down which was set to 1. Average data of three independent experiments ± S.D were shown. ** p<0.01, *** p<0.001.

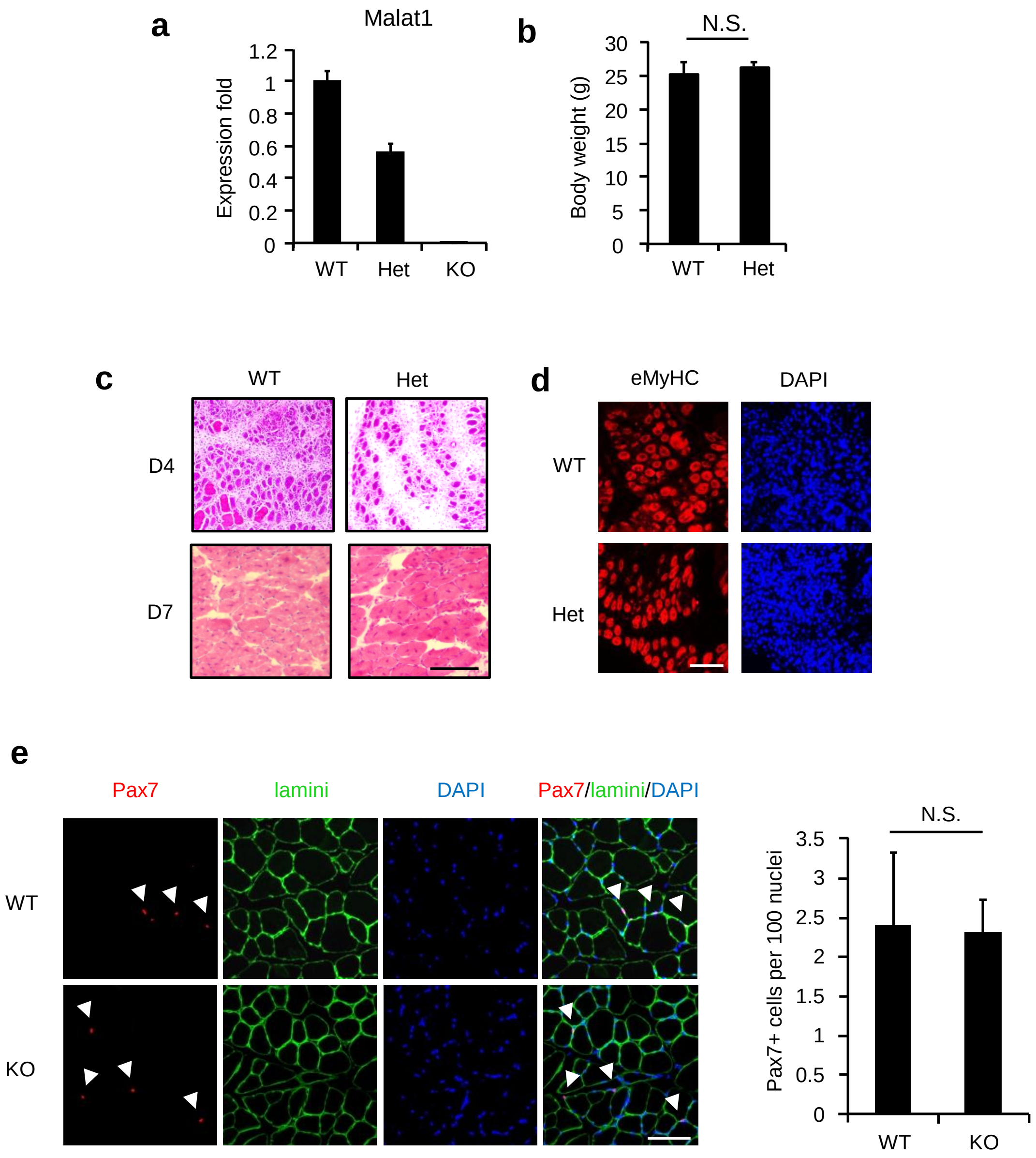

Supplementary Figure S5, Related to Figure 5. Heterozygous *Malat1* mice showed no acceleration in CTX induced muscle regeneration. (a) The expression levels of *Malat1* in WT, Heterozygous (Het) or Knockout (KO) mice were measured by qRT-PCR. (b) No significant difference in body weight was observed between WT and Het mice. Three pairs of littermates were used. (c) H&E staining of WT or Het TA muscle sections at day 4 or 7 after CTX injury revealed no significant difference in the degree of regeneration. (d) IF staining of eMyHC on the above muscle sections at day 4 revealed no significant difference. Scale bar=100 µm. (e) IF staining of Pax7 and lamini on the section of TA muscle from WT and KO mice. Scale bar=100 µm. Pax7 positive cells per 100 nuclei were calculated from 10 randomly chosen fields.

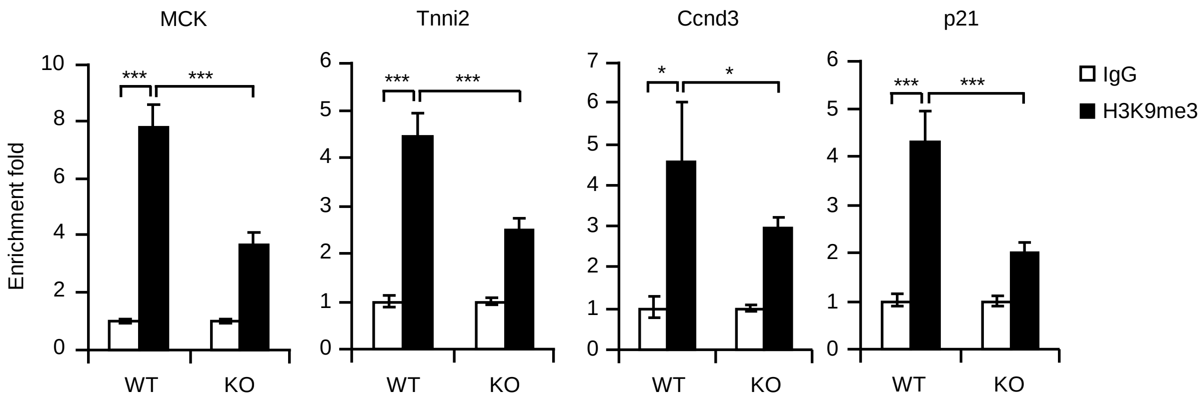

Supplementary Figure S6, Related to Figure 5. Depletion of *Malat1* in KO muscle decreased H3K9me3 enrichment on the MyoD binding loci. H3K9me3 enrichment on the MyoD binding loci, MCK, Tnni2, Ccnd3 and p21 promoters is decreased in KO vs WT muscles as assayed by ChIP-PCR in vivo. The data were represented as fold change compared to the level of IgG pull-down which was set to 1. Average data of three independent experiments ± S.D were shown. * $p<0.05$, *** $p<0.001$.

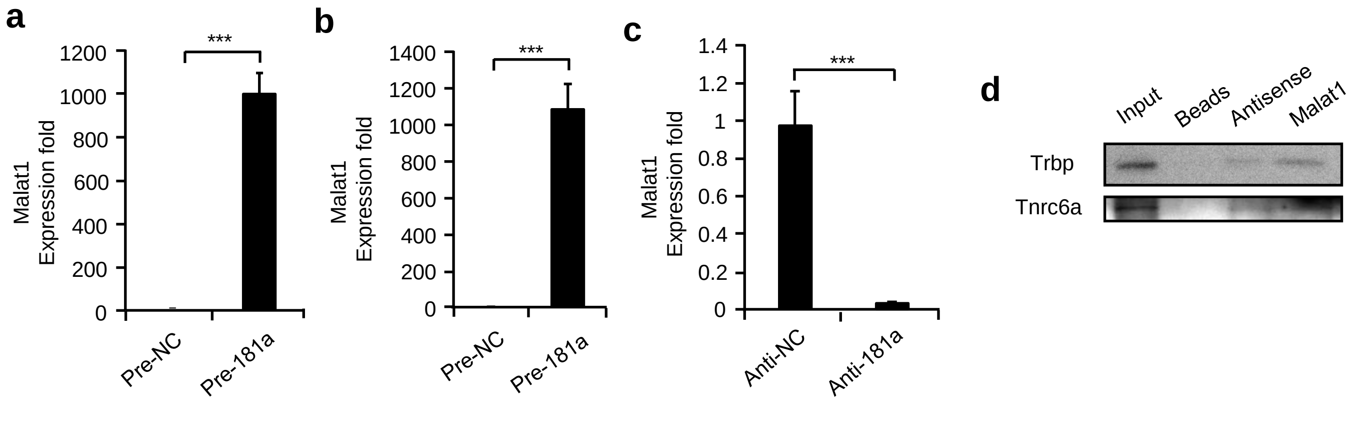

Supplementary Figure S7, Related to Figure 6. miR-181/nRISC regulates Malat1 expression. (a) The overexpression of miR-181a in C2C12 cytoplasm (a) or nucleus (b) after transfecting precursor miR181a (pre-181a) oligos was confirmed by qRT-PCR. (c) The knockdown of miR-181 by transfecting anti-miR-181a was confirmed by qRT-PCR. Average data of three independent experiments ± S.D were shown. *** $p<0.001$.(d) Biotinylated full length *Malat1* transcripts retrieved components of miRISC machinery including Trbp and Tnrc6a/GW182 from differentiating C2C12 nuclear lysates. As negative controls, beads only or antisense transcripts retrieved less amounts of proteins.
